# A Tumor-Homing Peptide Platform Enhances Drug Solubility, Improves Blood–Brain Barrier Permeability and Targets Glioblastoma

**DOI:** 10.3390/cancers14092207

**Published:** 2022-04-28

**Authors:** Choi-Fong Cho, Charlotte E. Farquhar, Colin M. Fadzen, Benjamin Scott, Pei Zhuang, Niklas von Spreckelsen, Andrei Loas, Nina Hartrampf, Bradley L. Pentelute, Sean E. Lawler

**Affiliations:** 1 Department of Neurosurgery, Brigham and Women’s Hospital, Harvard Medical School, Boston, MA 02115, USA; scottbj@alumni.beloit.edu (B.S.); pzhuang2@bwh.harvard.edu (P.Z.); niklas.von-spreckelsen@uk-koeln.de (N.v.S.); sean_lawler@brown.edu (S.E.L.); 2 Program in Neuroscience, Harvard Medical School, Boston, MA 02115, USA; 3 Harvard Stem Cell Institute, Harvard University, Boston, MA 02115, USA; 4 Broad Institute of MIT and Harvard, Cambridge, MA 02142, USA; blp@mit.edu; 5 Department of Chemistry, Massachusetts Institute of Technology, Cambridge, MA 02139, USA; farquhar@mit.edu (C.E.F.); cfadzen1@jh.edu (C.M.F.); loas@mit.edu (A.L.); nina.hartrampf@chem.uzh.ch (N.H.); 6 Department of General Neurosurgery, Centre of Neurosurgery, Faculty of Medicine and University Hospital, University of Cologne, 50937 Cologne, Germany; 7 Department of Chemistry, University of Zurich, Winterthurerstrasse 190, 8057 Zurich, Switzerland; 8 The Koch Institute for Integrative Cancer Research, Massachusetts Institute of Technology, Cambridge, MA 02142, USA; 9 Center for Environmental Health Sciences, Massachusetts Institute of Technology, Cambridge, MA 02139, USA; 10 Legorreta Cancer Center, Department of Pathology and Laboratory Medicine, Brown University, Providence, RI 02912, USA

**Keywords:** glioblastoma, peptide, brevican, drug targeting, precision medicine, chemotherapy, blood–brain barrier

## Abstract

**Simple Summary:**

Glioblastoma (GBM) is a fatal and incurable brain cancer, and current treatment options have demonstrated limited success. Here, we describe the use of a dg-Bcan-Targeting-Peptide (BTP-7) that has BBB-penetrating properties and targets GBM. Conjugation of BTP-7 to an insoluble anti-cancer drug, camptothecin (CPT), improves drug solubility in aqueous solution, retains drug efficacy against patient-derived GBM stem cells (GSC), enhances BBB permeability, and enables therapeutic targeting to intracranial patient-derived GBM xenograft in mice, leading to higher toxicity in GBM cells compared to normal brain tissues and prolonged animal survival. This work demonstrates a proof-of-concept for BTP-7 as a tumor-targeting peptide for therapeutic delivery to GBM.

**Abstract:**

Background: Glioblastoma (GBM) is the most common and deadliest malignant primary brain tumor, contributing significant morbidity and mortality among patients. As current standard-of-care demonstrates limited success, the development of new efficacious GBM therapeutics is urgently needed. Major challenges in advancing GBM chemotherapy include poor bioavailability, lack of tumor selectivity leading to undesired side effects, poor permeability across the blood–brain barrier (BBB), and extensive intratumoral heterogeneity. Methods: We have previously identified a small, soluble peptide (BTP-7) that is able to cross the BBB and target the human GBM extracellular matrix (ECM). Here, we covalently attached BTP-7 to an insoluble anti-cancer drug, camptothecin (CPT). Results: We demonstrate that conjugation of BTP-7 to CPT improves drug solubility in aqueous solution, retains drug efficacy against patient-derived GBM stem cells (GSC), enhances BBB permeability, and enables therapeutic targeting to intracranial GBM, leading to higher toxicity in GBM cells compared to normal brain tissues, and ultimately prolongs survival in mice bearing intracranial patient-derived GBM xenograft. Conclusion: BTP-7 is a new modality that opens the door to possibilities for GBM-targeted therapeutic approaches.

## 1. Introduction

High-grade gliomas, including grade III and grade IV glioblastoma (GBM), remain among the most difficult cancers to treat. GBM is the most common and deadliest primary malignant brain tumor, with a 5-year survival rate of only 5% [1]. Current standard-of-care which consists of surgery followed by chemotherapy and/or radiotherapy is non-curative, with almost all patients having recurrence with more aggressive tumors [2]. There is no standard-of-care for recurrent GBM. Malignant GBM cells invade into surrounding brain tissues, often escaping surgical resection and radiotherapy. Additionally, although a defining feature of GBM is abnormal angiogenesis leading to disorganized and leaky blood vessels, a significant subgroup of GBM cells are protected from chemotherapeutics by the blood–brain barrier (BBB) [3,4].

Most experimental GBM drugs from clinical trials over the past few decades have not made a major impact in limiting disease progression, and patients continue to suffer significant morbidity and death. The majority of therapies often fail clinically due to poor drug solubility [5], lack of tumor selectivity leading to undesired side effects [6], poor permeability across the blood–brain barrier (BBB), as well as extensive intra- and inter-tumor heterogeneity. The development of precision medicines with improved solubility and bioavailability, BBB penetrance, and the ability to target cancerous GBM cells selectively with minimal effect on healthy tissues would be a valuable strategy towards enhancing therapeutic efficacy. 

While GBM is well-known for cellular and genetic heterogeneity [7], the tumor extracellular matrix (ECM) has less spatial variability, making it an attractive target for therapeutic intervention strategies compared to specific membrane receptors that are only expressed on select tumor cell populations [8]. An ECM glycoprotein expressed exclusively in the central nervous system (CNS) called brevican (Bcan) is upregulated in GBM [9,10,11], and is linked to increased tumor invasion and aggressiveness [12]. The deglycosylated isoform, called dg-Bcan is only found in human high-grade glioma tissue samples [10,13], making it an attractive GBM-specific marker for the development of novel therapeutic targeting strategies. Previously, we have screened a combinatorial d-amino acid peptide library to identify an octameric dg-Bcan-Targeting Peptide, called BTP-7, that is stable in serum, binds dg-Bcan specifically, crosses the BBB, and preferentially homes to intracranial GBM xenografts in mice established using patient-derived GBM stem cells (GSC) [13]. Positron emission tomography (PET) imaging using [^18^F]-radiolabeled BTP-7 reveals higher tumor-to-brain uptake ratio (sustained over several hours) in comparison to values reported from similar studies evaluating clinical radiotracers such as [^18^F]FDG and [^18^F]FET [13]. These studies highlight BTP-7 as a promising tumor-specific agent for delivering functional cargoes to GBM, warranting further investigation and development. 

In this manuscript, we explore the potential of BTP-7 for the development of a targeting platform for GBM treatment. Camptothecin (CPT) is a potent topoisomerase I inhibitor that has shown promising results in preclinical models but failed to translate well in the clinic, mostly due to its insoluble nature [14,15]. Here, we show that chemical functionalization of CPT with BTP-7 (a positively charged peptide under physiological conditions) yields a peptide–drug conjugate (BTP-7-CPT) that is water-soluble and has BBB-penetrating properties. We demonstrate that BTP-7-CPT exhibits potency against patient-derived GBM stem cells in vitro, increases drug delivery to tumors in an intracranial patient-derived xenograft (PDX) mouse model of GBM resulting in enhanced tumor toxicity compared to healthy brain tissues, and ultimately prolongs survival in the animals.

## 2. Materials and Methods

### 2.1. Cell Culture

GBM-X6 is a xenoline that was established initially by direct implantation of freshly resected human GBM tissues into the flanks of immunocompromised athymic nude mice. GSCs are normally cultured and maintained as neurospheres in the absence of serum to preserve their stem-like state. GS401 is a GSC derived from a recurrent patient from Erasmus Medical Center [13]. We maintained both GBM-X6 and GS401 cultures in neurobasal (NB) supplemented with 2% B27, 1% glutamine, 20 ng/mL of fibroblast growth factor-2 (FGF), and 20 ng/mL of epidermal growth factor (EGF) [16]. Human embryonic kidney (HEK) and dg-Bcan-overexpressing HEK cells (HEK-Bcan) were cultured in DMEM supplemented with 10% FBS. All cells were cultured in a 37 °C incubator with 5% CO_2_ and 95% natural air.

### 2.2. Synthesis of BTP-7-CPT

Disulfide-cleavable CPT prodrug was synthesized as described [17]. The pyridyldithiol arm of the prodrug allows for conjugation to free thiols via disulfide exchange, enabling CPT to be attached to a cysteine residue on BTP-7. See SI for detailed synthesis.

### 2.3. Analysis of Compound Solubility

Dried powder of each compound was first resuspended in pure DMSO to form a 10 mm stock. Each stock was then diluted in saline (0.9% NaCl) to form an aqueous solution of 100 µm (final DMSO 1% (*v*/*v*)) or 1.3 mM (final DMSO 10% (*v*/*v*)) of each compound. The samples were incubated at room temperature for 5 min, then centrifuged at 10,000 rcf for 10 min to pellet any precipitates. 5 µL of supernatant was diluted into LC-MS vials containing 100 µL of 50:50 water:acetonitrile with 0.1% TFA additive and analyzed by LC-MS. The presence of soluble compound in each supernatant was determined by quantifying the ion count in the extracted ion chromatogram (EIC) using the MassHunter software. Solubility was measured by comparing the total EIC of the sample diluted in saline vs. in pure DMSO. Each sample was measured in duplicates. 

### 2.4. Western Blot Analysis

Western blotting was performed as previously described [13]. GBM-X6 and GS401 cells cultured as neurosphere suspensions were collected from the culture flask using a pipette. HEK293 and Bcan-overexpressing HEK293 (HEK-Bcan) cells cultured as adherent cells were harvested from their flask using a cell scraper. Cell lysates were extracted using NP40 cell lysis buffer, followed by sample separation on an SDS-PAGE gel, and then transferred onto a polyvinylidene difluoride (PVDF) membrane by Western blotting. The pan-brevican (Bcan) primary antibody [13] was used to detect the deglycosylated isoform of the Bcan protein. Horseradish peroxidase (HRP)-conjugated anti-rabbit IgG was used as a secondary antibody, and SuperSignal West Femto Maximum Sensitivity chemiluminescent substrate was used for enhanced chemiluminescence (ECL) detection. 

### 2.5. Cell Viability Assay

The CellTiter-Glo® luminescent cell viability assay was used. GBM-X6 and GS401 glioma stem cells [18] (cultured in Neurobasal growth media), or HEK293 cells (cultured in supplemented DMEM media) were washed once with PBS, dissociated to form single cell suspension using either StemPro Accutase for GBM-X6 and GS401 neurospheres, or 0.05% (*v*/*v*) Trypsin-EDTA for HEK293 cells, resuspended in the appropriate growth media, and then counted using a hemocytometer. Cells were seeded at the desired number (typically 30,000 GBM-X6 cells per well; 5000–20,000 HEK cells per well; 8000 GS401 cells per well) into clear-bottom black-well 96-well plates in triplicates. Stock solutions (10 mm) of CPT, BTP-7-CPT or scramble Scr-7-CPT were prepared in DMSO. A 1:3 serial dilution (in DMSO) of each stock was performed, and then 1 µL of each compound was added onto cells in each well containing 99 µL of media (in triplicate), so that a final concentration range of 0–100 µm (1% *v*/*v* DMSO) was achieved. Vehicle (DMSO with no drug) was added as a control. The plates were returned into a 37 °C tissue culture incubator. At every 24 h after incubation, a plate was removed from the incubator and 100 µL of CellTiter-Glo reagent was added to each well using a multi-channel pipette. The plate was incubated (in the dark) for 10 min on a nutating mixer, and then analyzed in a luminescence plate reader (POLARstar Omega, BMG Labtech, Offenburg, Germany). Each dataset was normalized to the vehicle (control), and plotted using GraphPad Prism. IC50 values were obtained using a non-linear fit (log(inhibitor) vs. response—variable slope (four parameters)). Each experiment was repeated 3 times to ensure reproducibility.

### 2.6. Permeability Analysis In Vitro Using Human BBB Organoids

Multicellular BBB organoids were established for 48 h as previously described [19,20]. Briefly, primary human astrocytes, primary human brain vascular pericytes (HBVP), and hCMEC/D3 brain endothelial cells were co-cultured in endothelial cell media (ECM) supplemented with 2% human serum in a sterile 96-well plate coated with 1% agarose. The cells were allowed to self-assemble into multicellular BBB organoids in a 37 °C incubator with 5% CO_2_ and 95% natural air for 48 h. The organoids were then pooled together in a microfuge tube and incubated with each drug conjugate at a final concentration of 10 μM in EGM containing 2% human serum for 3 h in a 37 °C incubator under constant rotation (*n* = 6–8 organoids per group). To examine if the drug conjugate affects the overall integrity of the BBB organoid surface, the organoids were also co-incubated with fluorescent dextran (TRITC-dextran (4.4 kDa)) at a final concentration of 10 μg/mL. The organoids were then washed three times with 1 mL of BBB working medium (see Supplementary Materials and Methods) and fixed in 3.7% formaldehyde in PBS for 10 min at room temperature. The organoids were then washed three times with 1 mL of media, transferred onto a thin-well chambered cover glass and imaged by confocal fluorescence microscopy. Quantification of peptide and dextran permeability was performed using the Fiji software, where the mean fluorescence intensity of the core of each organoid (at a depth between 50–90 μm) was measured and plotted using GraphPad Prism.

### 2.7. Ex Vivo Serum Stability Assay

Mouse serum was obtained from 8–10-week-old athymic female mice. Mice were euthanized by CO_2_ asphyxiation with secondary decapitation to ensure death. An incision was made from the neck to the abdomen and the thorax was cut to expose the heart. An amount of 100 µL of heparin was then injected directly into the right atrium to prevent the blood from clotting. A cardiac puncture was performed by piercing the left ventricle with a 21G needle to remove 400–1000 µL of blood. Blood from five mice was collected and combined in a 10-mL centrifuge tube and centrifuged for 5 min at 500 rcf at 4 °C. The top layer containing the serum (opaque) was removed and stored at −20 °C. 

To test for serum stability, a 10 mM stock of BTP-7-CPT and Scr-7-CPT was prepared in DMSO. Each compound (1.4 µL) was transferred into a microcentrifuge tube containing 200 µL of PBS with 25% mouse serum, or in PBS alone, and incubated at 37 °C (final concentration 70 μm). At timepoints 0, 1, 3, 6, and 12 h, 10 µL of each sample was removed from the microcentrifuge tube and transferred to a different microcentrifuge tube containing 20 µL of a solution of 2 M guanidine hydrochloride (GuHCl). Then, 10 µL of the sample from each time point was purified via solid-phase extraction with Millipore C18 10 µL ziptips to separate remaining peptide from serum proteins. Samples containing the peptide were eluted with 10 µL of 70% acetonitrile in water containing 0.1% TFA into an LC-MS vial containing 20 µL of water with 0.1% TFA additive and analyzed via LC-MS. The amount of intact compound was determined through quantifying the ion count in the extracted ion chromatogram (EIC) using the MassHunter software. Each timepoint was performed in duplicate. 

### 2.8. Intracranial GBM Implantation and Efficacy Studies

All animal protocols have been reviewed and approved by the Institutional Animal Care and Use Committee (IACUC). GBM-X6 GSC (100,000 cells) resuspended in 2 µL of PBS were inoculated into the right striatum of 6–8-week-old athymic female mice using a stereotactic frame as described. T2-weighted MRI of the brain was performed the day before treatment to ensure uniform tumor formation. The animals were then randomly assigned into groups and injected intraperitoneally (i.p.) with either BTP-7-CPT, Scr-7-CPT or vehicle control (DMSO in 0.9% NaCl) at 10 mg/kg (*n* = 6–7 mice). We employed either a late-stage treatment regimen (i.p. injection every 2 days from Day 25 to 49 post tumor implantation), or early-stage and longer treatment regimen (i.p. injection every 2 days from day 18 to day 68 post tumor implantation). At day 47 in the ‘late-stage’ therapy study, brains were imaged using T2-weighted MRI. Kaplan–Meier plot showing animal survival was graphed using GraphPad Prism.

### 2.9. Ex Vivo Immunofluorescence Staining of GBM Tissue Sections

At day 49 in the ‘late-stage’ therapy study, one mouse from each group was sacrificed by CO_2_ asphyxiation. The animals were perfused via transcardial perfusion with PBS, followed by 10% formalin. Then, the brains were excised, fixed in 10% formalin, frozen and cryosectioned into 16 µm sections for phospho-H2AX immunostaining. The rabbit anti-phospho-H2AX was used. Anti-rabbit Alexa-Fluor 647 secondary antibody (Invitrogen) and Hoechst dye were used for detection. Brain tissue sections were fixed with methanol for 1 min. The slides were then washed twice with PBS + 0.025% Triton X-100. Tissue sections were blocked with 10% normal goat serum diluted in PBS + 0.025% Triton X-100. Primary antibody against phospho-H2AX was added to the blocking solution (at 1:100 dilution) and the tissues were incubated overnight at 4 °C in a dark humidified box. The slides were then washed three times (2 min each) with PBS + 0.025% Triton X-100. Tissues were then incubated with Alexa Fluor secondary antibody and Hoechst 33,342 (both at 1:1000 dilution) in blocking solution. Then, the slides were washed three times (2 min each) with PBS + 0.025% Triton X-100. Vectashield mounting medium for fluorescence was applied onto the tissues, and a coverslip was mounted onto the slide. The edges of the coverslip were sealed with clear nail polish, and the tissues were imaged using an epifluorescence microscope under a 20× objective (*n* = 10).

### 2.10. Statistical Analysis

All datasets are presented as means ± SD. Significance is indicated on each graph and the statistical tests performed are indicated in the figure legends. The significance is represented as follows: * *p* < 0.05, ** *p* < 0.01, *** *p* < 0.001, **** *p* < 0.0001

### 2.11. Use of Human Specimens 

All patient-derived cells were obtained from patients who were undergoing surgical treatment at the Mayo Clinic in Rochester or Erasmus Medical Center. All subjects had provided informed consent and signed consent forms that were approved by the Institutional Review Board (IRB). Our lab received the cells in a de-identified manner.

## 3. Results

### 3.1. Conjugation of CPT with BTP-7 Enhances Drug Solubility

BTP-7 has a high isoelectric point of 10 and is soluble in aqueous solution [13]. Previously, we have observed that conjugation of Cy5.5, a hydrophobic fluorescent dye to BTP-7, enhances its water solubility [13]. As a proof-of-concept that BTP-7 functionalization improves the solubility of hydrophobic drugs, we examine here the physiochemical effect of conjugating BTP-7 to CPT, an insoluble chemotherapeutic [14,15]. 

#### 3.1.1. Synthesis of BTP-7-CPT

**Figure 1:**
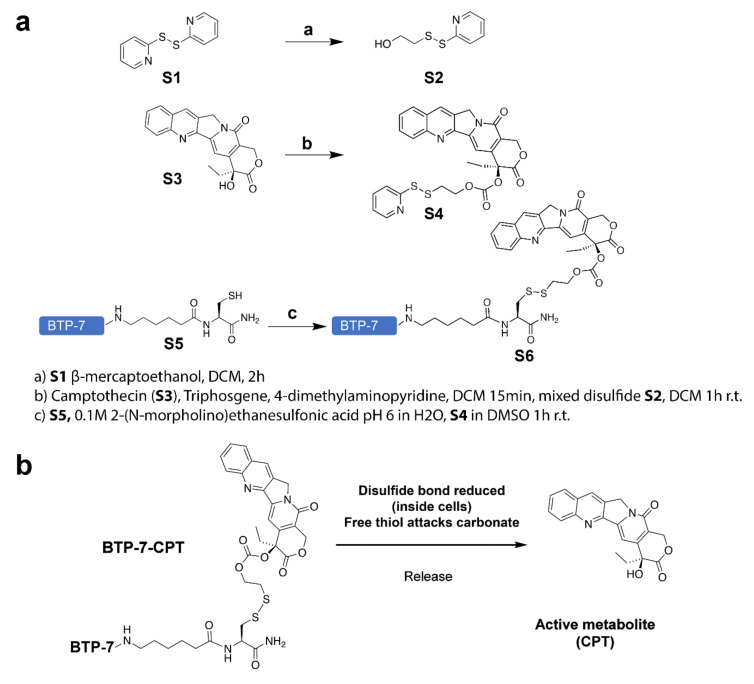
(**a**) Chemical synthesis workflow showing the conjugation of BTP-7 to camptothecin (CPT) (details listed in the SI); and (**b**) cleavage mechanism to release active CPT drug upon cell internalization.

BTP-7 containing an aminohexanoic acid linker (X) and a cysteine at the C-terminus was attached to a thiol on CPT via a previously reported disulfide linker [17] (workflow illustrated in Figure 1a). A detailed description of the synthetic route is provided as Supplementary Information. Within the reducing environment of the cellular cytosol, the disulfide linker is reduced to release the active CPT drug metabolite that is toxic to cells (Figure 1b) [21]. Using the same synthetic route, a scrambled version of the peptide was conjugated to CPT (Scr-7-CPT) as a control conjugate to assess for specific binding of the BTP-7 conjugate. 

#### 3.1.2. BTP-7 Conjugation Improves CPT Solubility 

To test the solubility of each compound in aqueous solution, a 10 mm stock of each compound in pure DMSO was diluted in saline solution (0.9% sodium chloride in water) to a final concentration of either 100 µm or 1.3 mm. The samples were incubated at room temperature for 5 min and centrifuged to remove any precipitate. The supernatant was analyzed by liquid chromatography–mass spectrometry (LC-MS). To quantify solubility, the extracted ion count from samples diluted in saline was normalized to samples diluted in pure DMSO.

**Figure 2:**
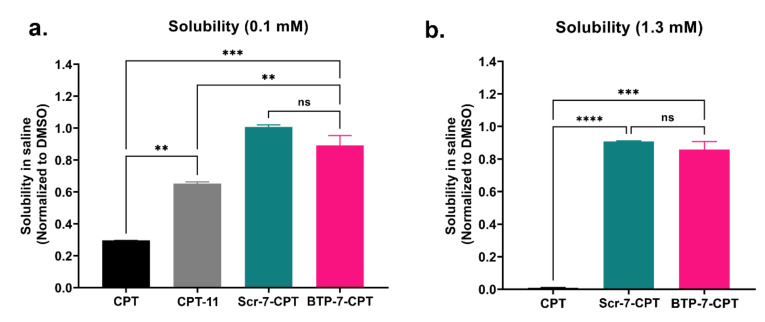
BTP-7 conjugation enhances drug solubility. (**a**) BTP-7 (and Scr-7) conjugation to CPT enhances drug solubility at 0.1 mM (low conc.) and (**b**) 1.3 mM (high conc.). CPT, CPT-11, BTP-7-CPT, and Scr-7-CPT stocks (in DMSO) were diluted in either DMSO or saline. Samples were centrifuged to pellet insoluble compound, and the resulting supernatant was analyzed by LC-MS. Solubility is measured by comparing the extracted ion chromatogram of the sample diluted in saline vs. in DMSO. Statistical significance was determined using a two-way ANOVA and Tukey’s multiple comparisons test (ns: non-significant, ** *p* < 0.01, *** *p* < 0.001, **** *p* < 0.0001).

As expected, the native unmodified CPT drug displayed low solubility, with 30% of soluble compound present in saline at 100 µm relative to in pure DMSO (Figure 2a and Figure S1a). CPT was practically undetectable in saline solution at a higher concentration of 1.3 mm (Figure 2b and Figure S1b). Conjugation of CPT to BTP-7 resulted in an increase in drug solubility at both concentrations (Figure 2 and Figure S1). Indeed, we demonstrated that BTP-7-CPT was more soluble than irinotecan (CPT-11) (Figure 2a and Figure S1a), a known water-soluble derivative of CPT that has been approved by the FDA for the treatment of advanced colorectal and pancreatic cancer [22]. The scramble Scr-7-CPT conjugate was also soluble in aqueous solution, similar to that of BTP-7-CPT (Figure 2 and Figure S1). This result indicates that the enhanced solubility is attributed to the intrinsic amino acid composition of the peptide, and not its specific sequence. 

### 3.2. BTP-7-CPT Binds dg-Bcan Protein

**Figure 3:**
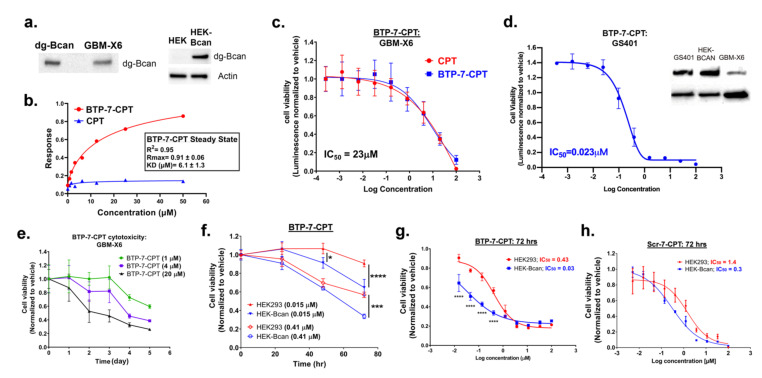
Cytotoxicity of BTP-7-CPT. (**a**) Western blot showing expression of dg-Bcan in patient-derived GBM-X6 cells, as well as in Bcan-overexpressing HEK cells (HEK-Bcan). (**b**) Binding kinetics analysis (steady-state) of BTP-7-CPT (red) and unmodified CPT (blue) to recombinant dg-Bcan protein using the ForteBio Octet system. R_max_ (maximal response in response unit) and K_D_ (dissociation constant) were calculated using a non-linear regression (one site specific binding) fit using the Graphpad Prism software. (**c**) Luminescent cell viability (CellTiter-Glo) of GBM-X6 cells treated with BTP-7-CPT (n_wells_ = 3) for 72 h. (**d**) Luminescent cell viability (CellTiter-Glo) of GS401 recurrent GBM cells treated with BTP-7-CPT (n_wells_ = 3) for 72 h. All IC_50_ values were measured through the non-linear ‘log(inhibitor) vs. response—variable slope (four parameters)’ fit. Western blot (right) shows higher expression of dg-Bcan in GS401 cells than in GBM-X6. (**e**) CellTiter-Glo assay of GBM-X6 cells treated with BTP-7-CPT at 1, 4, or 20 μM (n_wells_ = 3) over 5 days. (**f**) CellTiter-Glo assay of HEK293 (red) or Bcan-overexpressing HEK (HEK-Bcan) cells (blue) treated with BTP-7-CPT at 0.015, or 41 μM (n_wells_ = 3) over 3 days. (**g**,**h**) CellTiter-Glo assay of HEK-Bcan cells (blue) and control HEK293 cells (red) in the presence of (**g**) BTP-7-CPT or (**h**) Scr-7-CPT after 72 h. Statistical significance was calculated using a two-way ANOVA, Sidak’s multiple comparisons test (* *p* < 0.05, *** *p* < 0.001, **** *p* < 0.0001).

Next, we investigated BTP-7-CPT drug specificity and potency. Binding kinetics analyses using the Octet RED platform showed that BTP-7-CPT bound to purified recombinant dg-Bcan protein (K_D_ = 6.1 μM) (Figure 3b). This result is consistent with our previous observations which showed that BTP-7 was able to retain binding to dg-Bcan protein after being chemically linked to other molecules (i.e., a fluorophore, or radioisotope) [13]. As expected, unmodified CPT did not exhibit any affinity to recombinant dg-Bcan (Figure 3b). Altogether, these findings further highlight BTP-7 as a promising agent for therapeutic functionalization to enable dg-Bcan targeting. 

### 3.3. Cytotoxicity of BTP-7-CPT 

To evaluate BTP-7-CPT cytotoxicity in vitro, we used a well-characterized patient-derived xenoline (GBM-X6 cells) [18,23] and a recurrent GSC model (GS401 cells) [13]. Both GBM-X6 and GS401 cells express endogenous dg-Bcan (Figure 3a,d). Using the CellTiter-Glo luminescent cell viability assay, we showed that BTP-7-CPT was toxic to GBM-X6 cells with potency comparable with unmodified CPT (IC_50_ = 23 μM) (Figure 3c). The recurrent GS401 cells, which have higher dg-Bcan expression, were more sensitive to BTP-7-CPT cytotoxicity (IC_50_ = 0.023 μM) compared to GBM-X6 cells (Figure 3c,d). These suggest that chemically linking CPT to BTP-7 did not alter the drug’s potency, and that increased drug sensitivity is correlated with higher dg-Bcan expression, likely due to greater cellular uptake as previously demonstrated [13]. We further demonstrated that BTP-7-CPT was toxic to GBM-X6 cells in a dose- and time-dependent manner (Figure 3e). To investigate BTP-7-CPT specificity, we measured drug response in human embryonic kidney (HEK293) cells that do not express endogenous brevican, as well as in engineered HEK293 cells with stable brevican overexpression (HEK-Bcan) using the CellTiter-Glo assay. We found that both cell types responded to BTP-7-CPT in a dose- and time-dependent manner, although HEK-Bcan cells displayed higher sensitivity to BTP-7-CPT treatment compared to HEK293 cells (IC_50, HEK_ = 0.43 μM vs. IC_50, HEK-Bcan_ = 0.03 μM) (Figure 3f,g). No significant difference was observed with the scramble drug conjugate Scr-7-CPT (Figure 3h). These data further support the correlation of dg-Bcan expression in cells and drug sensitivity.

### 3.4. Analysis of BBB Permeability

**Figure 4:**
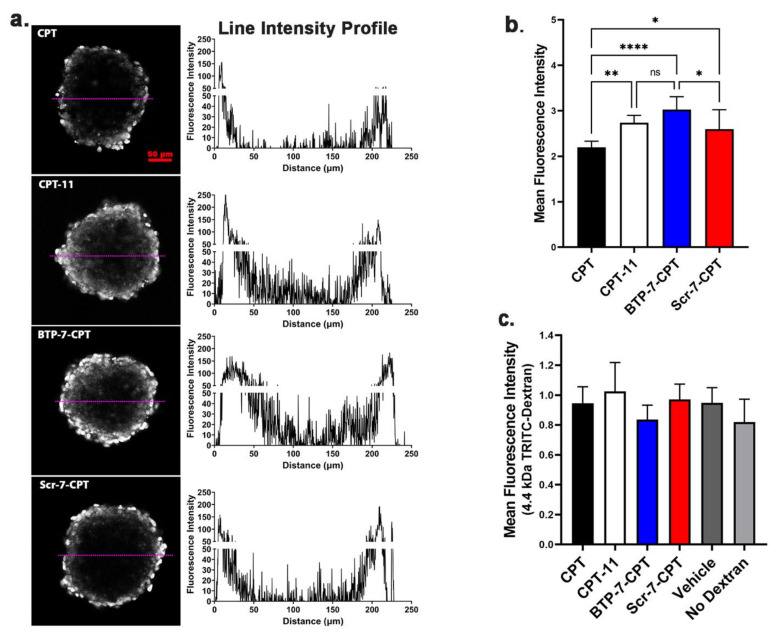
Conjugation of BTP-7 to CPT enhances permeability in human BBB organoids. (**a**) Fluorescence images showing the level of influx (penetration across the organoid surface) of BTP-7-CPT, Scr-7-CPT, CPT (camptothecin), and CPT-11 (irinotecan) (at 10 μM) in BBB organoids. Scale bar: 50 μm). Line profile (depicted by magenta line) showing the fluorescence (drug) level through the organoid. (**b**) Bar graph quantifying the mean fluorescence intensity of each drug conjugate, showing BTP-7-CPT had higher level of organoid influx compared to CPT and Scr-7-CPT (*n* = 8 organoids, *t* = 3 h). (**c**) Co-incubation of BBB organoids with each drug conjugate (from (**b**)) and 4.4 kDa TRITC-dextran (at 10 μg/mL) did not affect the level of dextran influx (all data were not significantly different compared to the vehicle control), indicating that the presence of the drug conjugate did not alter the organoid’s surface integrity (*n* = 6–8 organoids, *t* = 3 h). Statistical significance was determined using a one-way ANOVA and Tukey’s multiple comparisons test (* *p* < 0.05, ** *p* < 0.01, **** *p* < 0.0001).

We previously showed that BTP-7 conjugated to a fluorescent dye (Cy5.5) was able to cross the BBB in vitro using human BBB organoids and in vivo in mice [13]. To examine the potential of BTP-7 in enhancing CPT delivery across the BBB, we employed the in vitro human BBB organoid model [19,20]. CPT and its derivatives share a heterocyclic functional group that can be excited at 360 nm with fluorescence emission in the 440–700 nm range [24]. Using confocal fluorescence microscopy, we measured the level of influx of each drug conjugate into the BBB organoids. BTP-7-CPT displayed the highest influx into the organoids, comparable to that of CPT-11 which is known to cross the BBB (Figure 4a,b) [25]. Consistent with previous findings [13], the influx of the scramble Scr-7-CPT conjugate into the organoids was lower than that of BTP-7-CPT, but higher than unmodified CPT (Figure 4a,b). These data suggest that the enhanced BBB permeability observed is likely attributable to the intrinsic amino acid composition and/or structural properties of the peptide, although further studies to examine their specific mechanism of penetration are warranted. Finally, we demonstrated that the BBB organoids were able to restrict the entry of dextran (4.4 kDa) in the presence of these cytotoxic compounds (at 10 μM), indicating that the integrity of the organoids’ surface barrier remained intact for the duration of the experiment (Figure 4c).

### 3.5. Stability of Peptide–Drug Conjugate in Serum

**Figure 5:**
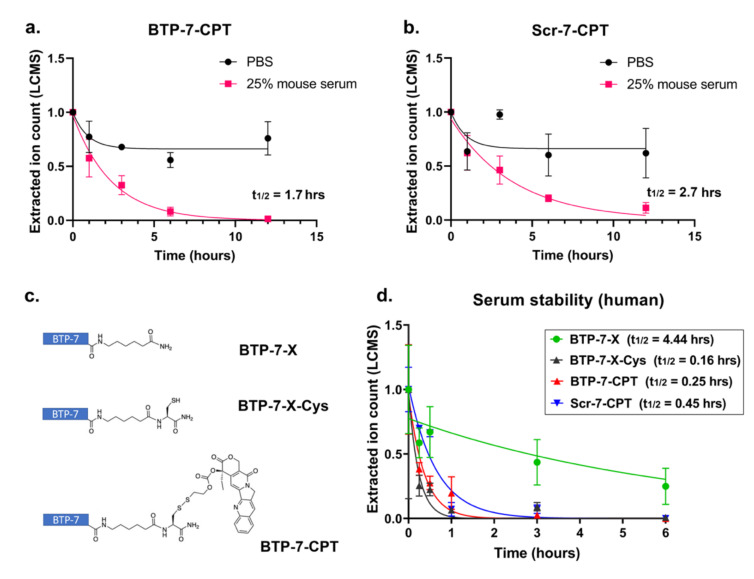
Stability of peptide–drug conjugates in serum. (**a**,**b**) LC-MS analysis showing the level of (**a**) BTP-7-CPT and (**b**) Scr-7-CPT detected (extracted ion count) in PBS and in 25% human serum over 12 h (*n* = 3). (**c**) Schematics of BTP-7 conjugated to an aminohexanoic acid linker (X) at the C-terminus (BTP-7-X), BTP-7-X functionalized with a cysteine (Cys) residue at the C-terminus (BTP-7-X-Cys), and BTP-7-CPT drug conjugate. (**d**) Stability of compounds from (**c**) in 25% human serum. All datasets were normalized to the EIC at *t* = 0 (*n* = 2).

Prior to animal testing, we performed ex vivo serum stability assays to measure the stability of BTP-7-CPT, Scr-7-CPT, and CPT in mouse serum. We have previously demonstrated that the native BTP-7 peptide (composed entirely of d-amino acids) linked to an aminohexanoic acid linker at the C-terminus (BTP-7-X) and the scrambled variant, Scr-7-X, were stable in mouse serum [13]. The unconjugated CPT drug was also stable in 25% mouse serum, with no decline in detectable compound over 12 h compared to in PBS (Figure S2). However, we observed that the concentration of both BTP-7-CPT and Scr-7-CPT compound declined over the same time frame in 25% mouse serum compared to in PBS, with the scramble Scr-7-CPT drug conjugate showing a slightly longer half-life (half-life_mouse_ = 2.7 h) compared to BTP-7-CPT (half-life_mouse_ = 1.7 h) (Figure 5a,b). We postulated that the source of BTP-7-CPT degradation in serum originated from the disulfide linkage connecting BTP-7 to CPT, which was primarily designed to be cleaved in the reducing environment of the cell to release the toxic CPT drug into the tumor. Indeed, we showed that while BTP-7-X was stable in 25% human serum, with approximately 40% of the peptide still intact after 24 h of incubation (half-life_human_ = 4.4 h) (Figure 5c,d) [13], the addition of a cysteine (Cys) residue to the C-terminus of BTP-7-X (BTP-7-X-Cys) resulted in a rapid decline of detectable compound (half-life_human_ = 0.16 h) (Figure 5c,d), likely due to the ability of Cys to form disulfides with serum proteins, which would result in the loss of BTP-7-X-Cys in the supernatant after the purification step to remove serum proteins. Similarly, we observed a relatively rapid decrease in BTP-7-CPT and Scr-7-CPT level in human serum (half-life_human_ = 0.25 and 0.45 h, respectively) compared to BTP-7-X (Figure 5c,d), suggesting compound instability that could be attributed to the likelihood of disulfide exchange occurring between the drug conjugates and serum protein.

### 3.6. Delivery of CPT to Intracranial GBM Tumor Using BTP-7

**Figure 6:**
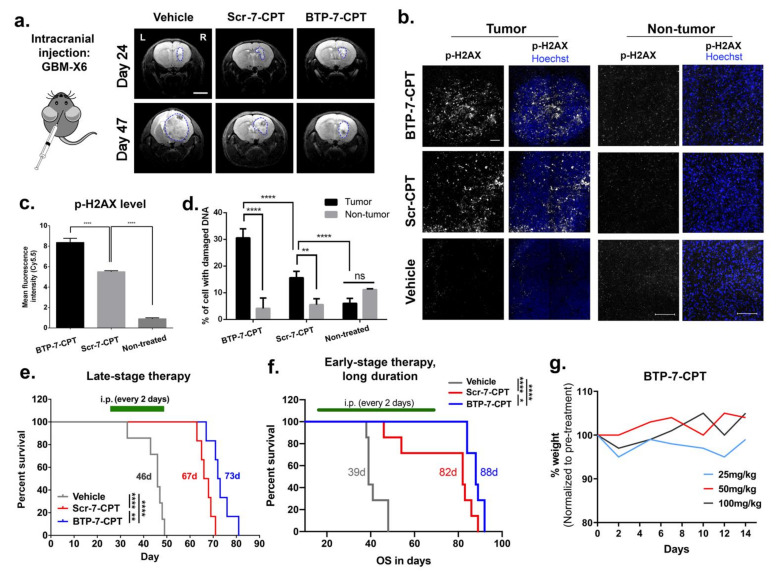
BTP-7 enhances drug delivery to GBM and prolongs survival in orthotopic PDX mouse model of GBM. (**a**) Representative MRI scans (coronal view) of mouse brain showing GBM-X6 (delineated with blue lines) after orthotopic xenotransplantation of patient-derived GBM-X6 cells in the right frontal lobe of the mouse brain at day 24 (before treatment) and day 47 (after treatment). Both the BTP-7-CPT and Scr-7-CPT groups showed reduced tumor burden compared to the control vehicle group. Scale bar: 2 mm. (**b**–**d**) Ex vivo analysis of brain cryo-sections from mice bearing GBM-X6 tumors after treatment. (**b**) Immunofluorescence staining for phospho-H2AX in tumor and non-tumor areas of mice brains harvested at day 49 after 13 treatments (nuclei stained with Hoechst dye (blue)). Scale bar: 100 microns. (**c**) Quantification of phospho-H2AX signal within the tumor areas shown in (**b**) (*n* = 3, one-way ANOVA and Tukey’s multiple comparisons test). (**d**) Quantification of the number of nuclei positive for phospho-H2AX signal in the tumor and non-tumor tissues of (**b**) (*n* = 3, two-way ANOVA and Sidak’s multiple comparison test (* *p* < 0.05, ** *p* < 0.01, **** *p* < 0.0001). (**e**,**f**) Kaplan–Meier survival plot of mice bearing GBM-X6 tumors that received (**e**) late-stage treatment (i.p.; 10 mg/kg dose starting at Day 25 to 49 post tumor implantation) or (**f**) early-stage and longer treatment duration (i.p.; 10 mg/kg dose starting from day 18 to day 68 post tumor implantation) with BTP-7-CPT (blue), Scr-7-CPT (red), or vehicle (gray). Treatment was performed every 2 days. In both studies, a significant difference (late-stage *p* < 0.0092; early-stage *p* < 0.022) is observed between the BTP-7-CPT and Scr-7-CPT treatment group, as well as between the drug conjugate and vehicle group (*p* < 0.0001) as determined by the Log-rank (Mantel–Cox) test (* *p* < 0.05, ** *p* < 0.01, **** *p* < 0.0001). (**g**) Graph showing the weights of the mice treated with BTP-7-CPT at 25, 50, and 100 mg/kg, indicating no decline in weight over more than 2 weeks.

To investigate BTP-7-CPT efficacy in an orthotopic GBM mouse model, a patient-derived GBM-X6 tumor xenograft was established in the right hemisphere of the frontal lobe of each mouse. Tumor formation was confirmed by MRI at day 24 post xenotransplantation (Figure 6a). At day 25, mice were randomized into three treatment groups to receive either vehicle (control), Scr-7-CPT, or BTP-7-CPT therapy (intraperitoneal (i.p.) 10 mg/kg, every 2 days). At day 47, we observed a decrease in tumor size in mice treated with BTP-7-CPT or Scr-7-CPT in comparison to vehicle (Figure 6a). Ex vivo analysis of brain cryo-sections showed that tumor tissues in both treatment groups displayed a higher level of phospho-H2AX (p-H2AX), indicating greater DNA damage in the treatment groups compared to the control (Figure 6b–d). As expected, tumor tissues from the BTP-7-CPT group had a higher level of p-H2AX than the Scr-7-CPT group, underscoring the ability of BTP-7 to improve drug targeting to GBM (Figure 6b–d). Importantly, both the treated groups showed negligible DNA damage in non-cancerous brain tissues from the left hemisphere of the brain (Figure 6b–d). 

Next, we evaluated their therapeutic efficacy using either a late-stage (day 25–49) or early-stage/long-duration (day 18–68) therapy intervention (i.p. 10 mg/kg, every 2 days). In both therapy regimens, treatment with BTP-7-CPT or Scr-7-CPT extended survival compared to the control animals that received only vehicle (Figure 6e,f). Mice treated with BTP-7-CPT showed survival benefit over mice treated with Scr-7-CPT (late-stage (73 vs. 67 days, *p* < 0.01); early-stage/long-duration (88 vs. 82 days, *p* < 0.05)) (Figure 6e,f). We further verified the safety of BTP-7-CPT by administering healthy nude mice with higher drug doses (25, 50, or 100 mg/kg). All animals showed no signs of moribundity and maintained their weight over 14 days, demonstrating that BTP-7-CPT is safe in mice even at higher therapeutic doses. Altogether, these results highlight that BTP-7-CPT is well-tolerated, preferentially targets patient-derived GBM xenograft in vivo without harming healthy brain tissues, and prolongs survival in mice. 

## 4. Discussion

Despite significant progress in understanding the molecular biology underlying GBM, few advancements have been achieved over the past two decades in the development of efficacious therapies for treating this morbid disease [26]. The poor prognosis and severe treatment side effects associated with GBM continue to drive efforts towards the development of new therapies. The precision medicine field has grown rapidly in recent years. However, clinical efficacy against GBM has been disappointing, mostly attributed to the significant inter- and intra-tumor genomic heterogeneity of GBM cells, limited delivery across the BBB, and the rapid development of resistant GBM phenotypes [27,28]. While many relevant genomic variants continue to be attractive targets, none of them have been strongly linked with clear prognostic and predictive value [6]. 

Our efforts are focused on developing a peptide platform targeting the deglycosylated isoform of Bcan (dg-Bcan) that is found exclusively in the ECM of all high-grade glioma (including GBM) tissues analyzed to date. This isoform is also distributed throughout the tumor [10,13]. The specificity, ubiquity, and accessibility of dg-Bcan in the GBM tumor microenvironment make it a very promising marker for the development of new targeted therapies. The biological roles of dg-Bcan remain to be fully investigated. Several studies have been reported on the major glycosylated isoform of the full-length Bcan protein that is expressed in normal CNS and is associated with synaptic stabilization and plasticity [29]. Bcan is upregulated and secreted by malignant gliomas, and is implicated in tumor invasion, progression, and poorer prognosis [30,31,32,33]. It has multiple isoforms produced by glycosylation, cleavage, and alternative splicing. However, only the dg-Bcan isoform is uniquely expressed in human high-grade gliomas and absent in non-cancerous adult brain tissues [34]. 

The mechanism of action of each Bcan isoform is poorly understood, although cleavage of the full-length Bcan is known to be necessary to promote glioma progression [35]. At the molecular level, Bcan and its cleaved products are secreted by the tumor cells, and activate EGFR/mitogen-activated protein kinase (MAPK) signaling and fibronectin production in glioma cells to enhance cell adhesion, migration, and invasive characteristics [30]. It is possible that the lack of glycosylation in dg-Bcan may regulate cleavage and signaling mechanisms, although these have not yet been fully elucidated. It is also not clear why dg-Bcan is retained on the cell surface without being released as a soluble protein [10,13]. However, this unique spatial expression provides an excellent and accessible target that is specific to GBM cells. Bcan is found to accumulate at the tumor invasion front [36]. It has been reported that while normal brain tissues exhibit radiation-induced downregulation of Bcan, GBM cells retain or upregulate Bcan expression after irradiation [37,38]. In another study, GBM tissues obtained from patients after receiving standard-of-care (maximal safe resection followed by radiotherapy/concomitant and maintenance temozolomide chemotherapy) and bevacizumab monotherapy upon tumor recurrence, exhibit high Bcan expression with no significant difference between the ‘poor’ or ‘better’ prognostic group [39]. Indeed, we have previously observed a high level of dg-Bcan in both the primary and corresponding recurrent patient-derived GSC cultures [13], further highlighting this protein as an important marker for targeting strategies against both the primary GBM tumor and the disease recurrence. The expression of Bcan following other forms of therapy (i.e., immunotherapy or oncolytic viral therapy) and its role in therapeutic resistance and GBM recurrence warrant further investigations.

Peptides are attractive tools for rationally designed targeting agents as they are small, cost-effective, scalable, and can be easily modified to select for desirable properties. With respect to GBM, this would include enhanced tumor specificity and BBB penetration [40,41]. They have significant potential for delivering therapeutics or diagnostics, while exhibiting faster diffusion and clearance rates [42]. BTP-7 is a small hydrophilic BBB-permeable peptide, and is made up entirely of d-amino acid residues which confer resistance to proteolytic degradation, altogether ensuing high peptide bioavailability in vivo. Clinical trials with CPT were discontinued in the 1970s due to its poor water solubility, high toxicity, and low response rate, leading to the development of CPT analogues with higher aqueous solubility (i.e., CPT-11) in the 1980s that were eventually approved by the FDA [43]. We found that BTP-7-CPT conjugate was even more soluble than CPT-11 (Figure 2a). Collectively, our data show that BTP-7 binds dg-Bcan specifically, is internalized by GBM cells, is able to penetrate the BBB, and enhances therapeutic targeting to GBM tumors leading to prolonged survival in a PDX mouse model of GBM [13].

In our efficacy studies in mice, we chose a treatment dose of 10 mg/kg, which is well below the highest dose tested (100 mg/kg) in our acute toxicity study, to ensure minimal risk of toxicity over continuous administrations. Overall, BTP-7-CPT treatment appears safe and well-tolerated in mice. Both BTP-7-CPT and its scramble counterpart Scr-7-CPT showed efficacy in our PDX GBM model compared to the control mice that received the vehicle, although BTP-7 enabled a higher level of drug targeting to the tumor compared to Scr-7, leading to increased tumor toxicity relative to healthy brain tissues. The Scr-7 peptide was initially devised to investigate the amino acid sequence specificity of BTP-7. However, we found that Scr-7-CPT also reached the tumor, which is not surprising given the enhanced permeability and retention (EPR) effect of GBM, the ability of Scr-7-CPT to cross the BBB (albeit less permeable than BTP-7) (Figure 4) [13], as well as higher Scr-7-CPT stability in mouse serum compared to BTP-7-CPT (Figure 5a,b). Nevertheless, the enhanced GBM-targeting properties of BTP-7-CPT compared to Scr-7-CPT offered some survival benefit in vivo. 

While native BTP-7 appears stable over 12 h in human serum [13], the BTP-7-CPT conjugate is completely degraded within 1 h (Figure 5d), suggesting a high likelihood of premature degradation of the disulfide linker in the bloodstream prior to reaching the tumor, despite this strategy having been successfully utilized for antibody–drug conjugates (ADC) in other studies [44]. Even though our previous pharmacokinetics analysis of [^18^F]BTP-7 radiotracer using PET imaging indicated that BTP-7 was almost immediately taken up by the GBM tumor post intravenous administration, future studies will aim to improve the linker stability in human serum as we continue to develop potential BTP-7-derived therapeutics for GBM therapy. Enzyme-labile peptide linkers cleaved through lysosomal hydrolysis upon cell internalization represent one potential option. Specifically, the valine–citrulline linker is cleaved by the lysosomal protease Cathepsin B that is upregulated in GBM [45]. This linker has been shown to have high serum stability and is used in several ADCs that are currently either in clinical trials or have been approved by the FDA [44]. Alternatively, ester linkers that are cleavable by esterases inside cells could be investigated, as they are considered stable cleavable linkers and have been employed for targeted delivery of CPT derivatives that are currently on the market [46]. We will also continue optimizing future therapeutic candidates based on the BTP-7 peptide platform for improved binding affinity to dg-Bcan, BBB permeability, and potency. 

## 5. Conclusions

We describe the design of the first BTP-7 therapeutic conjugate and show that BTP-7 could provide a robust platform for targeting various anti-cancer therapeutics to GBM. In addition to potent small molecules, other payloads that may be delivered with our platform include drug-loaded nanoparticles, radionuclides for targeted radiotherapy, or even potent lead candidate drugs that may have previously failed in clinical trials due to poor solubility. Taken together, BTP-7 is a new modality that opens the door to possibilities for targeted therapeutic approaches for GBM.

## 6. Patents

Partners Healthcare Innovation has supported the filing of a provisional patent application (# 62/739845, 1 October 2018) and a full US patent application (# 17/282,028, 1 April 2021), in which the core claim is the composition of matter directed towards a novel brain-tumor-targeting molecule, BTP-7, and an accompanying method of use claims set to said composition in brain tumor therapeutic applications such as GBM treatment and brain tumor imaging. 

## Data Availability

All data generated from this study are available in the manuscript or the Supplementary Materials.

## Supplementary Materials

The following supporting information can be downloaded at: https://www.mdpi.com/article/10.3390/cancers14092207/s1, Figure S1: Drug solubility in aqueous solution. Figure S2. Stability of CPT drug in mouse serum. Full images of western blots can also be found here.
