# Supplementary material for: A Tumor-Homing Peptide Platform Enhances Drug Solubility, Improves Blood–Brain Barrier Permeability and Targets Glioblastoma
Source: Cancers (Basel). 2022 Apr 28;14(9):2207. doi: 10.3390/cancers14092207 (PMC9103942; doi:10.3390/cancers14092207)
Supplement: Supplementary file 1 [file cancers-14-02207-s001.zip › cancers-1633186-supplementary.pdf]

Supporting Information

Title: A tumor-homing peptide platform enhances drug solubility, improves blood-brain-barrier permeability and targets glioblastoma

Authors: Choi-Fong Cho[1,2,3,4]*, Charlotte E. Farquhar[5], Colin M. Fadzen[5], Benjamin Scott[1], Niklas von Spreckelsen[1,6], Andrei Loas[5], Nina Hartrampf[5,7], Bradley L. Pentelute[4,5,8,9], Sean E. Lawler[1,10]

This Supporting Information file includes:

Supplementary Materials and Methods
Supplementary Figures S1 and S2
Supplementary Notes: Synthesis route, sequences, and chromatograms
Supplementary Full Western Blots

Supplementary Materials and Methods

Chemicals and reagents:

For peptide synthesis, N^{α}-Fmoc protected D-amino acids (a.a., Fmoc-D-Ala-OH, Fmoc-D-Arg(Pbf)-OH; Fmoc-D-Asn(Trt)-OH; Fmoc-D-Asp-(O*t*-Bu)-OH; Fmoc-D-Cys(Trt)-OH; Fmoc-D-Gln(Trt)-OH; Fmoc-D-Glu(O*t*-Bu)-OH; Fmoc-D-His(Boc)-OH; Fmoc-D-Ile-OH; Fmoc-D-Leu-OH; Fmoc-D-Lys(Boc)-OH; Fmoc-D-Lys(alloc)-OH, Fmoc-D-Met-OH; Fmoc-D-Phe-OH; Fmoc-D-Pro-OH; Fmoc-D-Ser(*t*-But)-OH; Fmoc-D-Thr(*t*-Bu)-OH; Fmoc-D-Trp(Boc)-OH; Fmoc-D-Tyr(*t*-Bu)-OH; Fmoc-D-Val-OH) and Fmox-L-Lys(biotin)-OH were purchased through Advanced ChemTech (Louisville, KY). Chem-Impex (Wood Dale, IL) and Peptides International (Louisville, KY). N^{α}-Fmoc protected L-amino acids (Fmoc-L-Ala-OH, Fmoc-L-Arg(Pbf)-OH; Fmoc-L-Asn(Trt)-OH; Fmoc-L-Asp-(O*t*-Bu)-OH; Fmoc-L-Cys(Trt)-OH; Fmoc-L-Gln(Trt)-OH; Fmoc-L-Glu(O*t*-Bu)-OH; Fmoc-Gly-OH, Fmoc-L-Ile-OH; Fmoc-L-Leu-OH; Fmoc-L-Lys(Boc)-OH; Fmoc-L-Met-OH; Fmoc-L-Phe-OH; Fmoc-L-Pro-OH; Fmoc-L-Ser(*t*-But)-OH; Fmoc-L-Thr(*t*-Bu)-OH; Fmoc-L-Trp(Boc)-OH; Fmoc-L-Tyr(*t*-Bu)-OH; Fmoc-L-Val-OH;) were purchased from the Novabiochem-line through Millipore Sigma (Darmstadt, Germany). Fmoc-L-His(Boc)-OH was bought from CEM. Succinimidyl 4,4'-azipentanoate (NHS-Diazirine) was purchased from Thermo Fisher Scientific (Waltham, MA) (#26167). Amino acids in peptide sequences are abbreviated with one letter code; capitalized letters refer to L-amino acids, lowercase letters refer to D-amino acids. 6-aminohexanoic acid, Irinotecan (CPT-11), and (S)-(+)-Camptothecin were purchased from ChemImpex (Wood Dale, IL).

H-Rink Amide-ChemMatrix resin was obtained from PCAS BioMatrix Inc. (St-Jean-sur-Richelieu, Quebec, Canada). 4-pentynoic acid, Fmoc-L-propargylglycine, 2-(1H-benzotriazol-1-yl)-1,1,3,3-tetramethyluronium hexafluorophosphate (HBTU), and 2-(7-aza-1H-benzotriazole-1-yl)-1,1,3,3-tetramethyluronium hexafluorophosphate (HATU) were purchased

from Chem Impex (Wood Dale, IL) and P3 Biosystems (Louisville, KY). (7-Azabenzotriazol-1-yloxy)tripyrrolidinophosphonium hexafluorophosphate (PyAOP) was purchased from P3 Biosystems (Louisville, KY). AldraAmine trapping agents (for 1000–4000 mL DMF), N-methyl pyrrolidinone (NMP), triisopropylsilane (TIPS), *t*-butylmethyl ether (TBME), Diisopropylethylamine (DIEA; 99.5%, biotech grade), piperidine (ACS reagent, ≥99.0%), trifluoroacetic acid (HPLC grade, ≥99.0%), triisopropylsilane (≥98.0%), formic acid (FA, ≥95.0%), dimethyl sulfoxide (DMSO, HPLC grade, ≥99.7%) were purchased from Sigma-Aldrich. N,N-Dimethylformamide (DMF), dichloromethane (DCM), and HPLC-grade acetonitrile were from EMD Millipore (Billerica, MA). All solvents used for HPLC-MS were purchased from EMD and Fluka (Darmstadt, Germany). Cy5.5-azide was obtained from Lumiprobe (Hallandale Beach, FL). All other chemicals and reagents were purchased from Sigma-Aldrich (St. Louis, MO). Water was deionized using a Milli-Q Reference water purification system (EMD Millipore, Billerica, MA). Nylon 0.22 μm syringe filters were TISCH brand SPEC17984.

The following molecular biology supplies and reagents were used in our experimental studies: Recombinant human brevican (R&D Systems Minneapolis, MN; Cat. # 4009-BC-050), Streptavidin-coated pink-fluorescent magnetic particles (2.0–2.9 μm, Spherotech, Lake Forest, IL; Cat. # FSVM-2058-2), *O*-glycosidase (Roche; 11347101001), Neuraminidase (Sialidase) (Sigma-Aldrich, St. Louis, MO); Cat. # 10269611001), PNGase F (New England Biolabs, Ipswich, MA; Cat. # P0704S), Octet Ni-NTA Biosensors (Forte Bio, Fremont, CA; Cat. # 18-5101), CellTiter-Glo 3D cell viability assay (Promega, Madison, WI; Cat. # G983); Heparin Solution (STEMCELL Technologies; Cat. # 07980); Phospho-H2AX (Cell Signaling Technology; Cat. # 9718), Hoechst dye (Life Technologies; Cat. # H3570), Horseradish peroxidase (HRP)-conjugated anti-rabbit IgG (GE Healthcare; Cat. # NA934V), SuperSignal

West Femto Maximum Sensitivity chemiluminescent substrate (Thermo Fisher Scientific; Cat. # 34096), clear-bottom black-well 96-well plates (Greiner BIO-ONE; Cat. # 655090).

Cell lines and culture conditions

GBM stem cells (GSC) GBM-X6 were cultured as neurospheres in Neurobasal medium (Invitrogen, Carlsbad, CA) supplemented with 2% B27 (Invitrogen), 1% glutamine (Invitrogen), epidermal growth factor (EGF) (20 ng/ml; PeproTech, Rocky Hill, NJ), and fibroblast growth factor-2 (FGF) (20 ng/ml; PeproTech, Rocky Hill, NJ). The GBM-X6 cells used in our studies had been passaged once in a mouse brain, where the cells were implanted intracranially and allowed to form a solid tumor over 30 days. Then, the GBM-X6 tumor was excised, dissociated in a tissue culture flask and cultured in Neurobasal medium as above. Human embryonic kidney (HEK) cells was cultured in DMEM (Invitrogen, Carlsbad, CA) supplemented with 10% FBS. Cells were grown in T25 or T75 vented-cap tissue culture flasks (Sarstedt AG and Co). HEK-Bcan cells were generated by cloning hBCAN cDNA into the pcDNA3.1 vector and stably transfected in HEK293 cells. Primary human astrocytes were purchased from Lonza Bioscience, Basel, Switzerland and cultured in Astrocyte Growth Medium (AGM; Lonza Bioscience, Basel, Switzerland) (consisting of astrocyte basal medium supplemented with hEGF (human epidermal growth factor), insulin, ascorbic acid, GA-1000 (Gentamicin, Amphotericin-B), L-glutamine and 1% fetal bovine serum (FBS)). Astrocytes were grown in T75 Cell+ vented-cap tissue culture flasks (Sarstedt AG and Co, Nümbrecht Germany). Human brain microvascular pericytes (HBVP) (ScienCell Research Laboratories, Carlsbad, CA) were cultured in Pericyte Medium (ScienCell Research Laboratories, Carlsbad, CA) containing 2% FBS, pericyte growth supplement and penicillin-streptomycin. Immortalized human cerebral microvascular endothelial cells (hCMEC/D3) (Cedarlane Laboratories, Burlington, Canada) were maintained in culture in Endothelial Growth Medium

(EGM-2) containing hEGF, hydrocortisone, GA-1000, FBS, VEGF, hFGF-B, R^3-IGF-1, ascorbic acid and heparin (Lonza Bioscience, Basel, Switzerland). For BBB organoid formation in low-attachment co-culture condition and functional assays, the organoids were maintained in EGM-2 (Lonza Bioscience, Basel, Switzerland) supplemented with 2% human serum (Valley Biomedical, Winchester, VA; Cat. # HS1021) and with the elimination of VEGF supplementation (this media formulation will henceforth be known as 'BBB working media'). All cells were cultured in a humidified incubator at 37 °C with 5% CO_2, and 95% natural air. For cell dissociation, StemPro Accutase Cell Dissociation Reagent (Thermo Fisher Scientific, Waltham, MA; Cat. # A1110501) was used on GSC cultured as neurospheres, and Trypsin-EDTA (0.05% v/v, with phenol red) (Thermo Fisher Scientific, Waltham, MA; Cat. # 25300054) was used for detaching adherent cells. All cell lines were regularly tested for mycoplasma contamination.

Instruments:

For fluorescence imaging, the Zeiss LSM 710 laser scanning confocal microscope or the Nikon Eclipse Ti epi-fluorescence microscope equipped with a QIClick camera was used. For wide-field time-lapse imaging for cell invasion/migration assays, the Nikon Eclipse TE2000-U epi-fluorescence microscope was used. For binding kinetic analyses, the Octet RED384 Platform (Forte Bio) from the Harvard University Center for Molecular Interactions was used.

Liquid chromatography–mass spectrometry (LC-MS)

For mass spectrometry analysis, the filtered peptide solution (10 μL of a 1mg/mL solution) was diluted in 50% acetonitrile in water with 0.1% TFA (90 μL) to a final concentration of approximately 0.1 mg/mL. LCMS chromatograms and associated high resolution mass spectra were acquired using an Agilent 6520 Accurate-Mass Q-TOF LCMS (abbreviated as 6520) or

an Agilent 6550 iFunnel Q-TOF LCMS system (abbreviated as 6550). Solvent compositions used in the LCMS are water with 0.1% formic acid additive (solvent A) and acetonitrile with 0.1% formic acid additive (solvent B).

Mass-directed reversed-phase high performance liquid chromatography (RP-HPLC)

For RP-HPLC purification, the crude lyophilized peptides were dissolved in water with 0.1% TFA additive containing a minimal amount of acetonitrile for solubility (e.g. 5% acetonitrile). All samples were filtrated through a Nylon 0.22 µm syringe filter prior to purification. For all HPLC purifications, a gradient of acetonitrile with 0.1 % TFA additive (solvent B) and water with a 0.1% TFA additive (solvent A) was used unless otherwise noted. Specific purification conditions such as column and gradient are specified for each case.

NMR Spectroscopy. Proton nuclear magnetic resonance (^{1}H NMR) spectra were recorded in 5 mm tubes on a Bruker Avance Neo spectrometer in deuterated solvents at room temperature. Chemical shifts (δ scale) are expressed in parts per million (ppm) and are calibrated using residual protic solvent as an internal reference (DMSO: δ = 2.50 ppm). Data for ^{1}H NMR spectra are reported as follows: chemical shift (δ ppm) (multiplicity, coupling constants (Hz), integration). Couplings are expressed as: *s* = singlet, *d* = doublet, *t* = triplet, *q* = quartet, *m* = multiplet or combinations thereof. Carbon chemical shifts (δ scale) are also expressed in parts per million (ppm) and are referenced to the central carbon resonance of the solvent (DMSO: δ = 39.52 ppm). In order to assign the ^{1}H and ^{13}C NMR spectra, a range of 2D NMR experiments (COSY, HSQC, HMBC, NOESY) were used as appropriate.

Infrared spectroscopy (IR). Infrared spectra (IR) were recorded on a Bruker Alpha II FTIR. IR data is reported in frequency of absorption (cm^{-1}). The IR bands are characterized as: *w* = weak, *m* = medium, *s* = strong, *br* = broad, or combinations thereof.

Octet binding kinetic analysis

The FortéBio OctetRed384 was used to study the binding kinetics of each peptide to recombinant human brevican (in PBS and 0.1 mM EDTA, pH 6.8). The brevican protein was deglycosylated prior to experimental use as detailed above. All binding kinetics assays were performed within the OctetRed instrument under agitation at 1000 rpm in 0.9% NaCl irrigation with 0.05% Tween (working buffer). Assays were performed at 30°C in solid black 384-well plates (Geiger Bio-One). The final volume for all the solutions was 80 µl/well. Firstly, Ni-NTA biosensors were soaked for 10 min in working buffer. Before loading the protein onto each biosensor, a baseline was established in working buffer for 60 s. Deglycosylated brevican, dg-Bcan (50 µg/ml; His-tagged) was loaded on the surface of each biosensor for 180 s. Typical capture levels varied slightly between 0.5 and 2 nm, and variability within run did not exceed 0.1 nm. Reference biosensors were exposed to the 'de-glycosylation buffer' lacking brevican protein during the loading step as internal controls. A 60 s biosensor washing step was applied. Then, biosensors were exposed to the analyte (peptide) in working buffer (ranging between 0–10 µM BTP) for 300 s during the 'association' step. Finally, the biosensors were exposed to working buffer (without peptide) during the 'dissociation' step for 600 s. Binding affinity of each peptide was assessed through steady state analysis, where the response unit was plotted over peptide concentration. All data was plotted and curves were fitted using the non-linear 'one-site specific binding' fit, and the dissociation constant (K_D) value was calculated using the GraphPad Prism software.

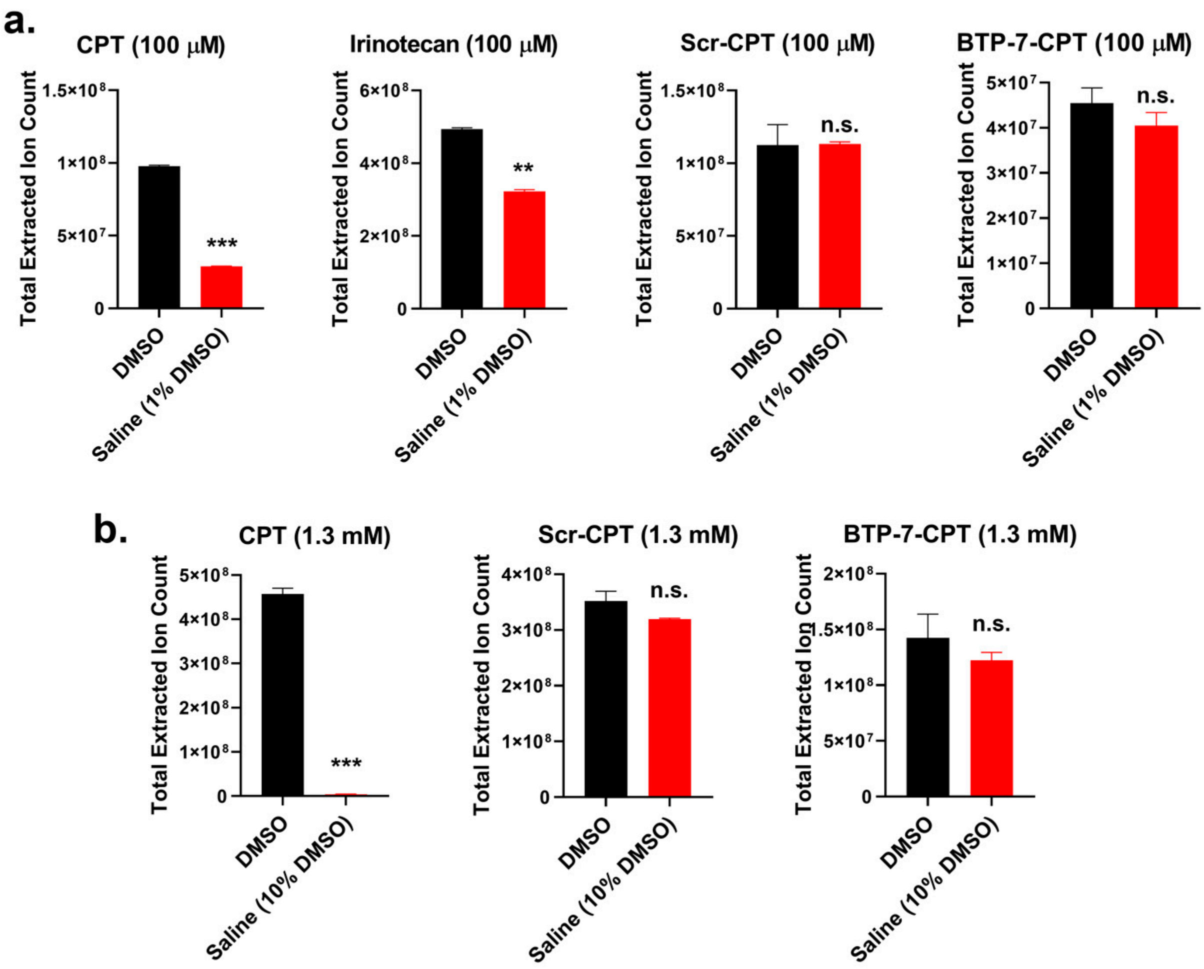

Figure S1. Drug solubility in aqueous solution. a,b) Drug solubility of compounds diluted in saline vs. in pure DMSO at either **(a)** low concentration (100 μM) or **(b)** high concentration (1.3 mM). Samples were centrifuged to pellet insoluble compound, and the resulting supernatant analyzed by liquid chromatography mass spectrometry (LCMS). Solubility is measured by comparing the extracted ion count of the sample diluted in saline vs. in DMSO. Statistical significance was determined using the Student's t-test.

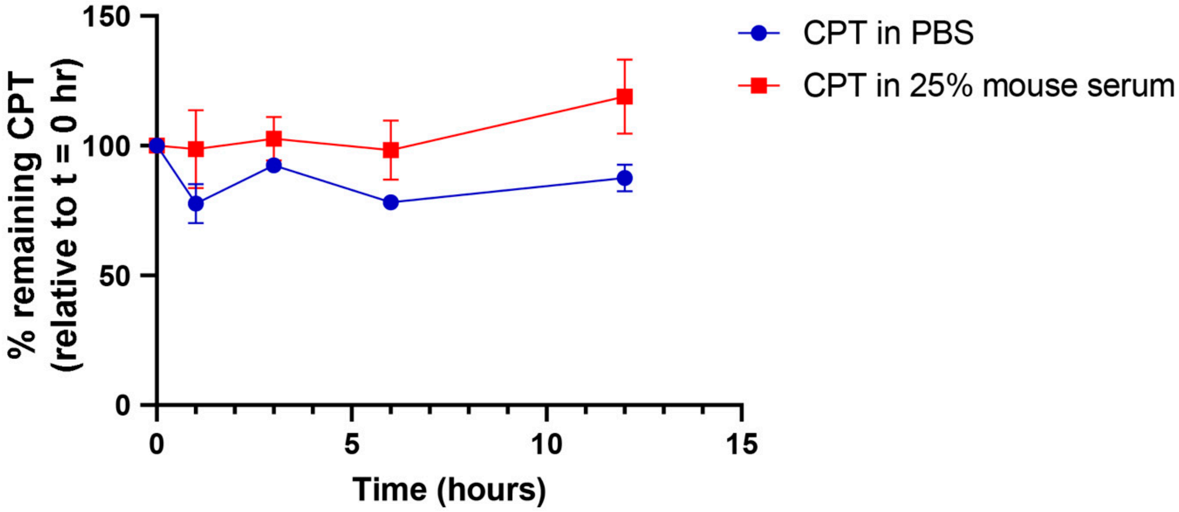

Figure S2. Stability of CPT drug in mouse serum. Percentage of CPT detected (extracted ion count) in PBS or 25% human serum over 12 hrs as determined by LC-MS. Each dataset was normalized to the extracted ion count at $t = 0$ ($n = 2$).

Supplementary Notes:

Synthesis of Camptothecin-linker and conjugation to BTP-7

2-(pyridin-2-yldisulfaneyl)ethan-1-ol (S2)[1]

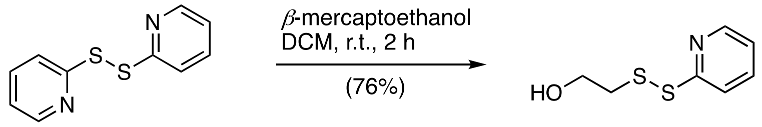

S1 **S2**

2-Mercaptoethanol (500 µL, 7.10 mmol, 1 eq.) and 2,2'-dipyridyl disulfide (**S1**, 4.70 g, 21.4 mmol, 3 eq.) were dissolved in DCM (20 mL) and stirred at room temperature for 3 hrs. Afterwards, the reaction mixture was concentrated under reduced pressure and flash column chromatography [hexanes/EtOAc, 10:1 to 2:1] afforded mixed disulfide **S2** (1.01 g, 5.39 mmol, 76%) as a yellow solid.

$\mathbf{R}_f$ = 0.45 [hexanes/ethyl acetate, 1:1].

^{1}H NMR(500 MHz, DMSO-d_6) δ 8.50 – 8.42 (m, 1H), 7.88 – 7.75 (m, 2H), 7.24 (ddd, J = 6.6, 4.8, 2.1 Hz, 1H), 4.99 (t, J = 5.5 Hz, 1H), 3.62 (q, J = 6.1 Hz, 2H), 2.92 (t, J = 6.3 Hz, 2H) ppm.

^{13}C NMR (500 MHz, DMSO-d_6) δ 159.5, 149.5, 137.8, 121.1, 119.3, 59.1, 41.2 ppm.

FTIR (thin film): $\tilde{\nu}$ = 3288 (br), 2920 (w), 2863 (w), 1574 (m), 1415 (s), 1043 (m), 754 (s) cm^{-1}.

HRMS (ESI): calcd. for $C_7H_{10}NOS_2^+$: 188.0198 [M+H]$^+$
found: 188.0269 [M+H]$^+$.

2-pyridinyldithioethyl carbonate Camptothecin (S4)[1]

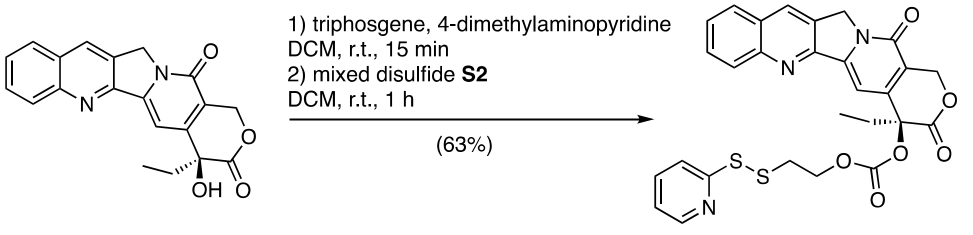

S3 **S4**

Camptothecin (**S3,** 250 mg, 0.718 mmol, 1 eq.), triphosgene (82.6 mg, 0.278 mmol, 0.387 eq.) and 4-dimethylaminopyridine (459 mg, 3.75 mmol, 5.21 eq.) were combined in dry DCM (10 mL), and after 15 min, mixed disulfide **S2** was added (148 mg, 0.790 mmol, 1.10 eq.) and the reaction was stirred at room temperature for 4 hrs. Flash column chromatography [DCM:Acetone, 20:1 to 4:1] afforded **S4** (254 mg, 0.452 mmol, 63%).

R$_f$ = 0.27 [hexanes/ethyl acetate, 1:3].

^{1}H NMR (500 MHz, DMSO-d_6) δ 8.68 (s, 1H), 8.39 (ddd, J = 4.8, 1.9, 0.9 Hz, 1H), 8.18 – 8.09 (m, 2H), 7.85 (ddd, J = 8.5, 6.8, 1.5 Hz, 1H), 7.81 – 7.71 (m, 1H), 7.71 – 7.63 (m, 2H), 7.15 (ddd, J = 7.4, 4.8, 1.1 Hz, 1H), 7.09 (s, 1H), 5.52 (d, J = 2.0 Hz, 2H), 5.29 (s, 2H), 4.33 (t, J = 6.0 Hz, 2H), 3.20 – 3.08 (m, 2H), 2.25 – 2.13 (m, 2H), 0.92 (t, J = 7.4 Hz, 3H) ppm.

^{13}C NMR (500 MHz, DMSO-d_6) δ 167.1, 158.6, 156.5, 152.7, 152.2, 149.6, 147.9, 146.3, 144.7, 137.7, 131.6, 130.4, 129.8, 129.0, 128.5, 128.0, 127.8, 121.3, 119.4, 119.2, 94.4, 77.9, 66.5 66.8, 50.3, 36.8, 30.3, 7.6 ppm**.**

FTIR (thin film): $\tilde{\nu}$ = 2992.83 (w), 1748.22 (m), 1667.56 (m), 1748.22 (m), 1667.56 (m),1253.34 (m), 666.08 (s) cm^{-1}.

HRMS (ESI): calcd. for $C_{28}H_{23}N_3O_6S_2^{+}$: 562.1101 [M+H]$^+$;
found: 562.1104 [M+H]$^+$.

BTP-7-Camptothecin (S6)

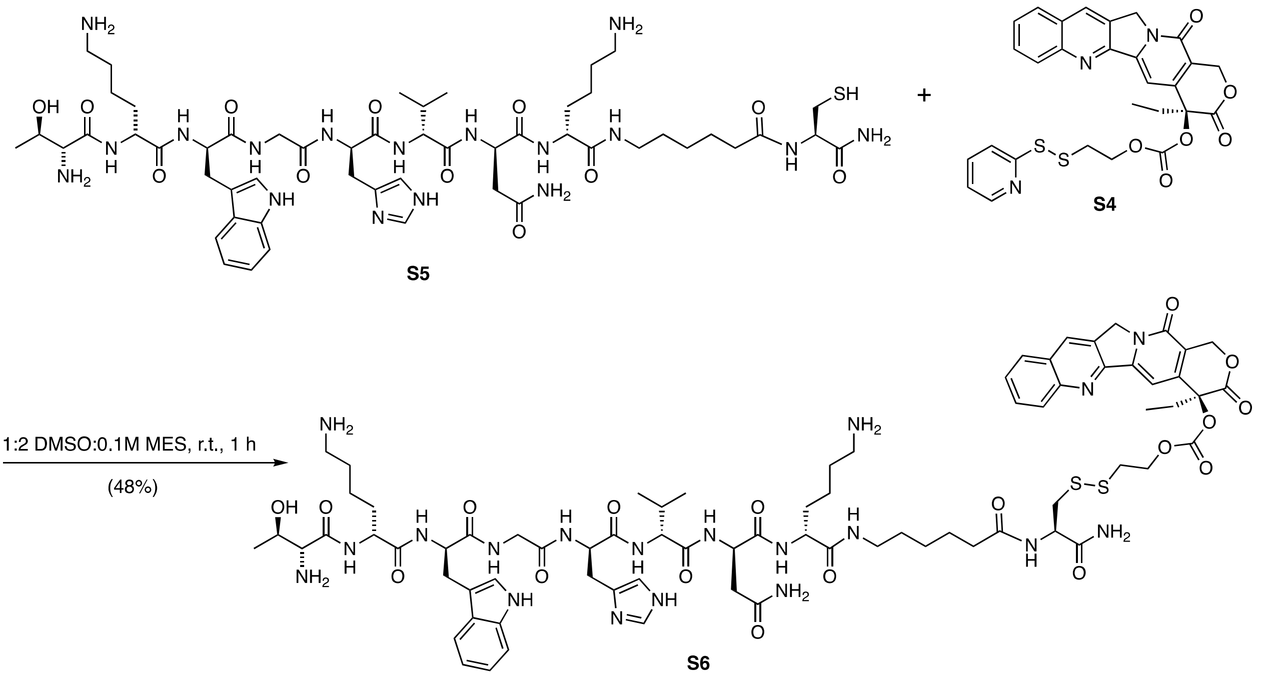

The pyridyldithiol arm of **S4** allows for conjugation to free thiols via disulfide exchange, enabling **S4** to be attached to BTP-7 with a C-terminal cysteine **S5**. To perform this conjugation, the peptide (**S5**, 50 mg, 32 µmol, 1.9 eq.) in 2-(N-morpholino)ethanesulfonic acid (MES, 0.1 M, pH 6, 14 mL) was combined with **S4** (9.5 mg, 17 µmol, 1 eq.) in DMSO (7 mL) and stirred at room temperature for 1 hr. The reaction was quenched with trifluoroacetic acid (0.20 mL, 2.6 mmol, 150 eq.). The camptothecin-

BTP-7 (**S6**) conjugate was purified by RP-high-performance liquid chromatography (RP-HPLC) in 100 mM ammonium acetate buffer (pH = 5) and an acetonitrile gradient (15–30% over 75 min) (15 mg, 8.1 µmol, 48%). Purity of each RP-HPLC fraction was confirmed by LC-MS.

HRMS (LC-MS): calcd. for $C_{76}H_{103}N_{19}O_{18}S_2^+$: 817.8661 $[M+2H]^{2+}$
found: 817.8666 $[M+2H]^{2+}$.

Scrambled BTP-7-Camptothecin (S8)

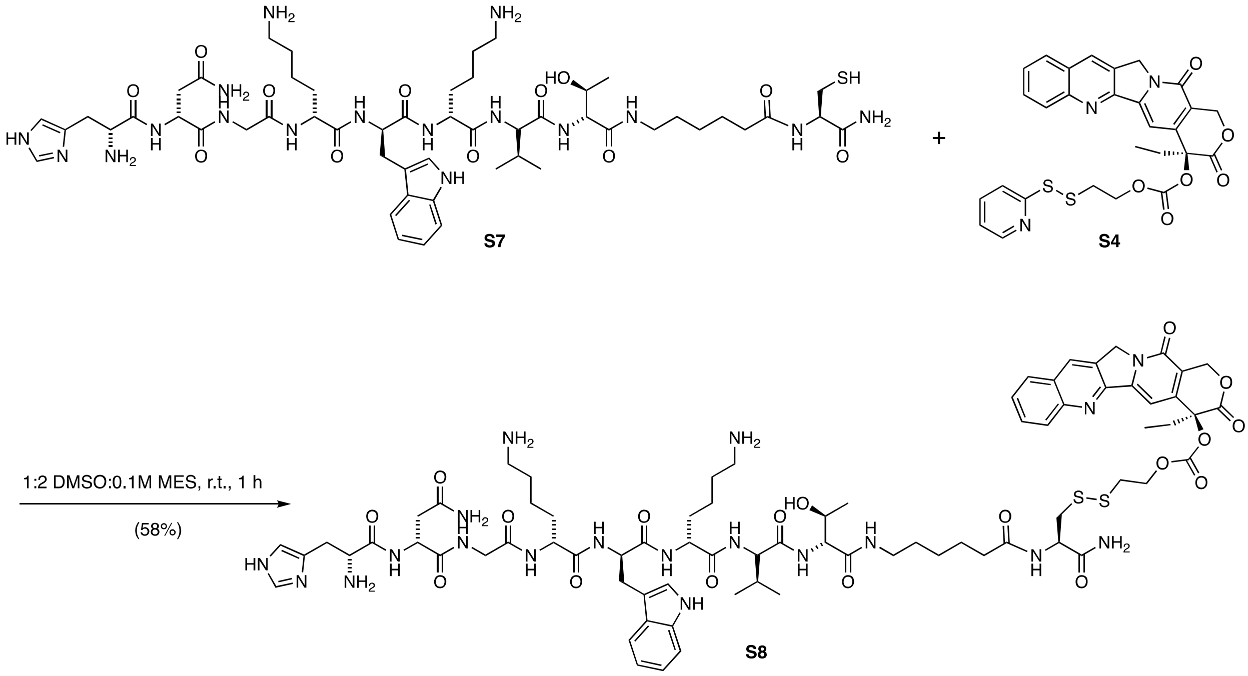

The conjugation and purification protocols given for **S6** were used to conjugate the scrambled BTP-7 peptide (**S7**) to camptothecin to synthesize the camptothecin-scramble (**S8**) (18 mg, 9.7 µmol, 58%).

HRMS (LCMS): calcd. for $C_{76}H_{103}N_{19}O_{18}S_2^+$: 817.8661 $[M+2H]^{2+}$
Found: 817.8669 $[M+2H]^{2+}$.

2-(pyridin-2-yldisulfaneyl)ethan-1-ol (S2)

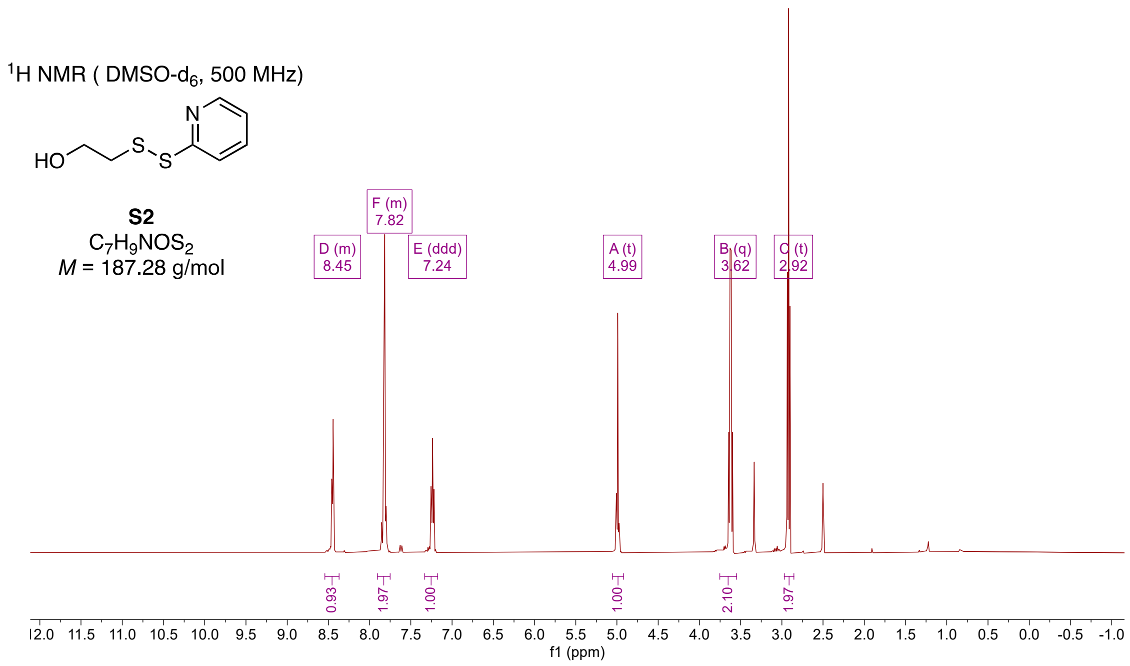
1H NMR (DMSO-d6, 500 MHz)
N
HO
S
S
S2
C7H9NOS2
M = 187.28 g/mol
F (m)
7.82
D (m)
8.45
E (ddd)
7.24
A (t)
4.99
B (q)
3.62
C (t)
2.92
0.93
1.97
1.00
1.00
2.10
1.97
12.0 11.5 11.0 10.5 10.0 9.5 9.0 8.5 8.0 7.5 7.0 6.5 6.0 5.5 5.0 4.5 4.0 3.5 3.0 2.5 2.0 1.5 1.0 0.5 0.0 -0.5 -1.0
f1 (ppm)

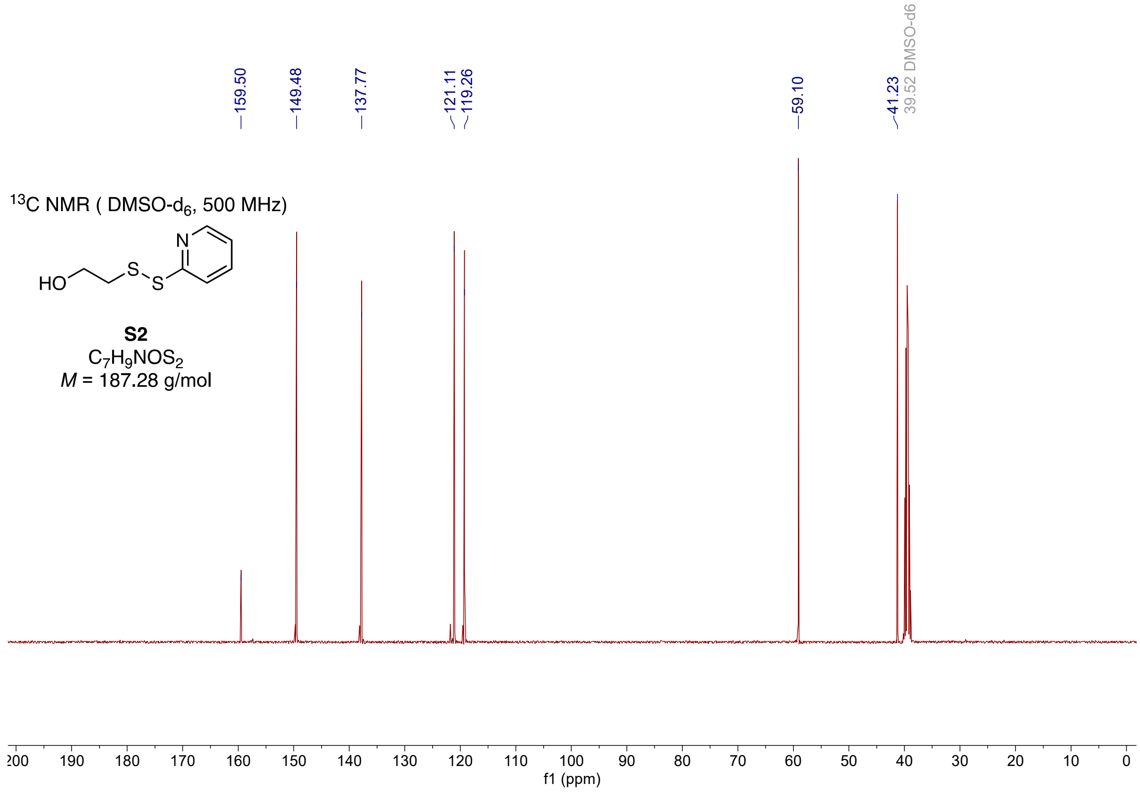
159.50
149.48
137.77
121.11
119.26
59.10
41.23
39.52 DMSO-d6
13C NMR (DMSO-d6, 500 MHz)
N
HO
S
S
S2
C7H9NOS2
M = 187.28 g/mol
200 190 180 170 160 150 140 130 120 110 100 90 80 70 60 50 40 30 20 10 0
f1 (ppm)

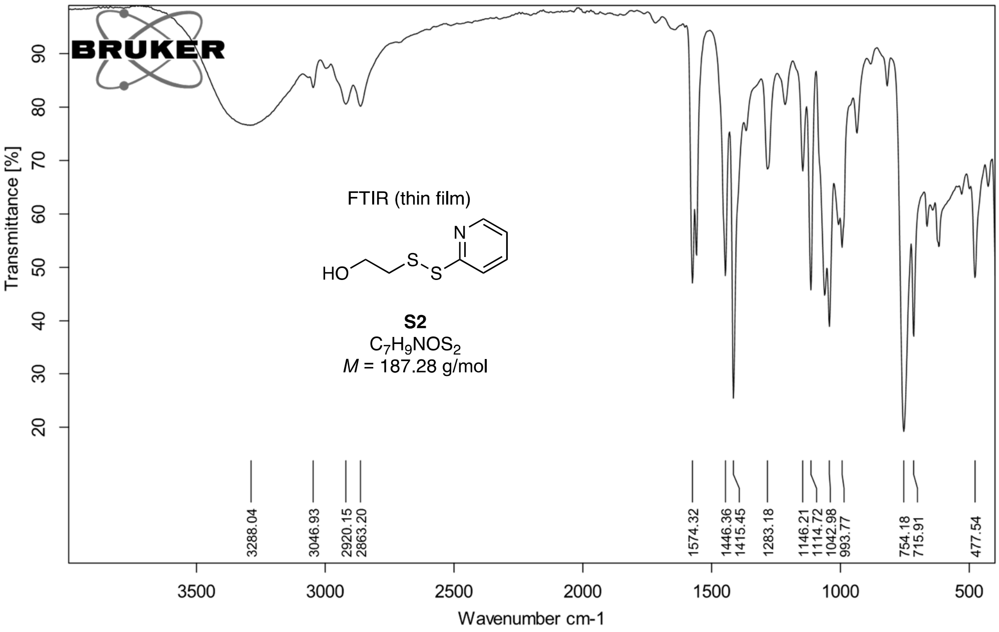
BRUKER
Transmittance [%]
FTIR (thin film)
HO
S
S
N
S2
$C_7H_9NOS_2$
M = 187.28 g/mol
3288.04
3046.93
2920.15
2863.20
1574.32
1446.36
1415.45
1283.18
1146.21
1114.72
1042.98
993.77
754.18
715.91
477.54
Wavenumber cm-1

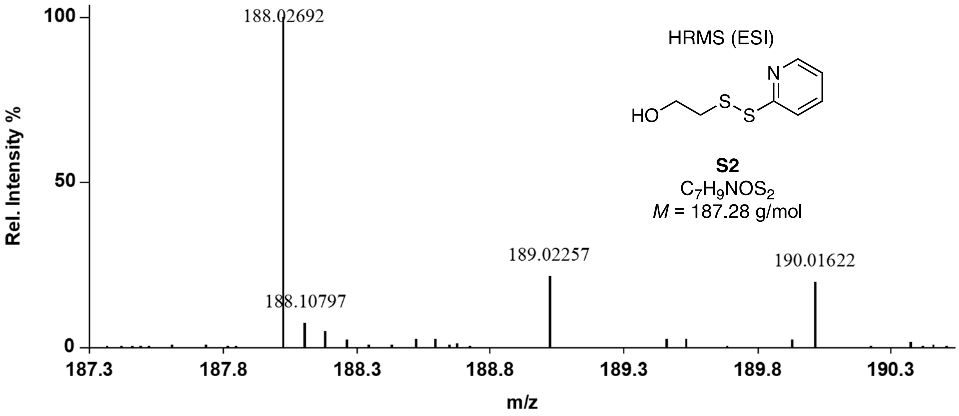
Rel. Intensity %
188.02692
HRMS (ESI)
HO
S
S
N
S2
$C_7H_9NOS_2$
M = 187.28 g/mol
189.02257
190.01622
188.10797
m/z

2-pyridinyldithioethyl carbonate Camptothecin (S4)

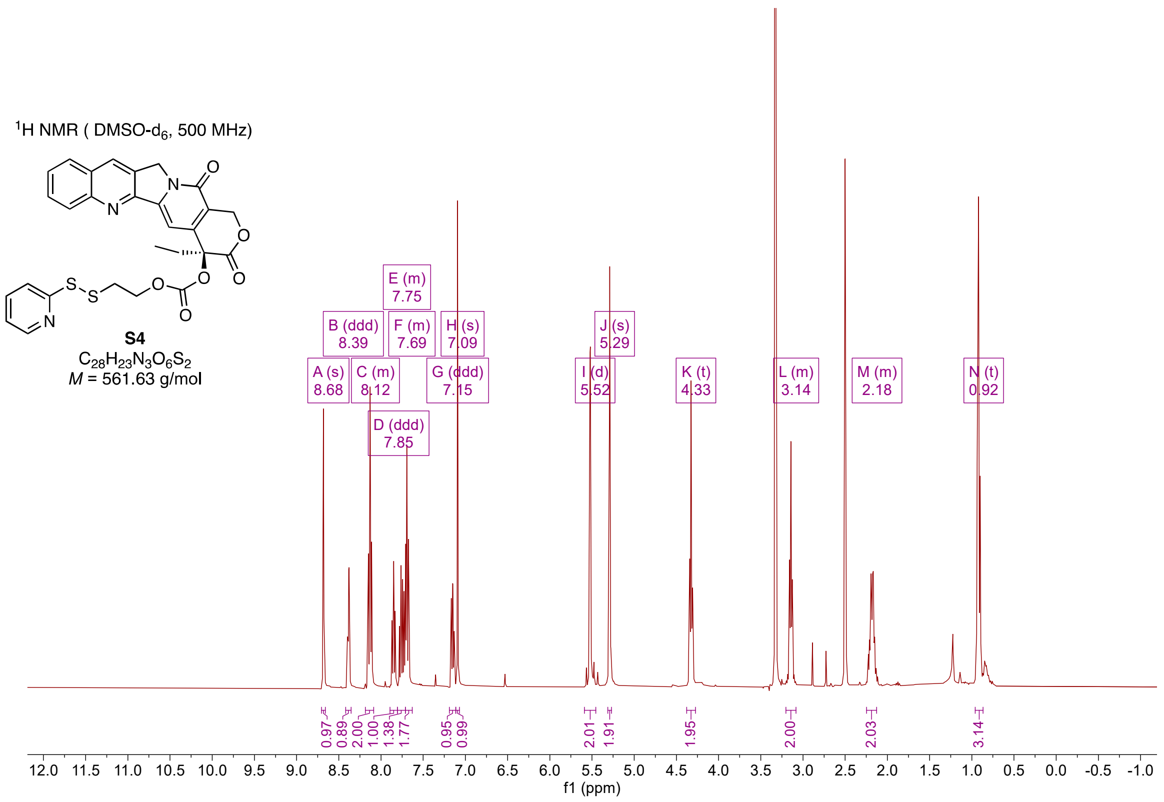

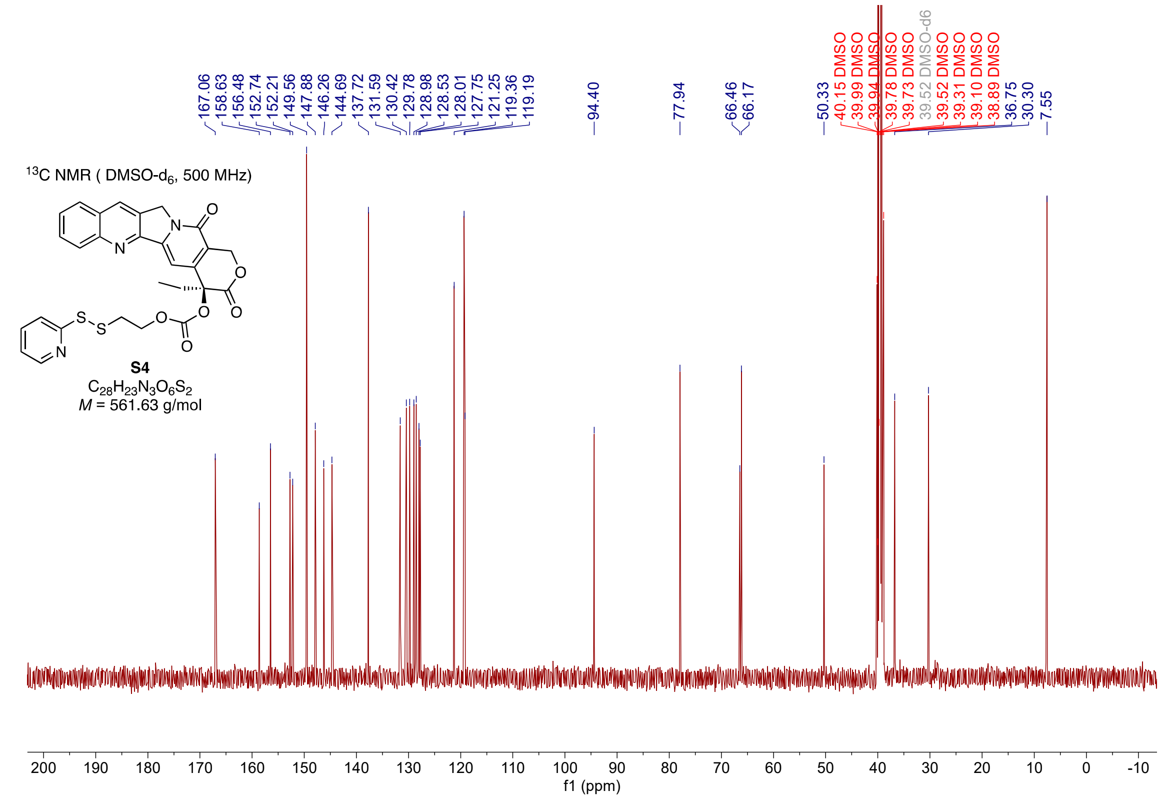

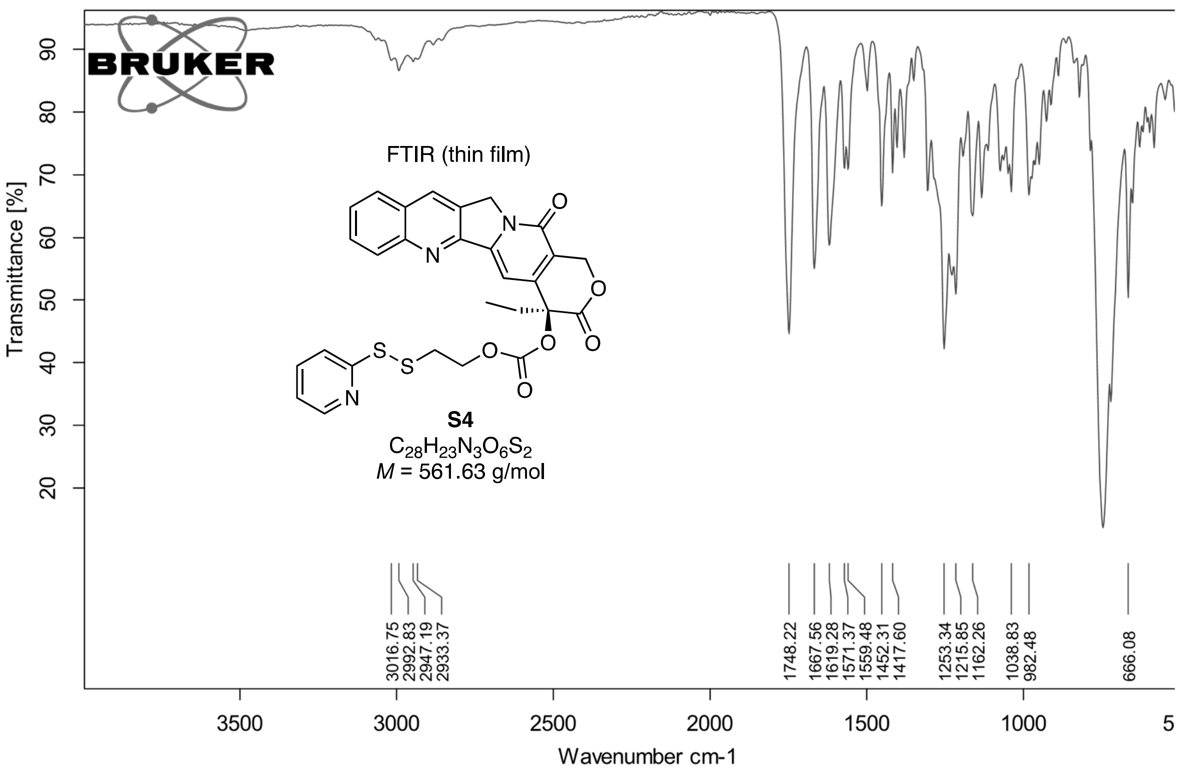

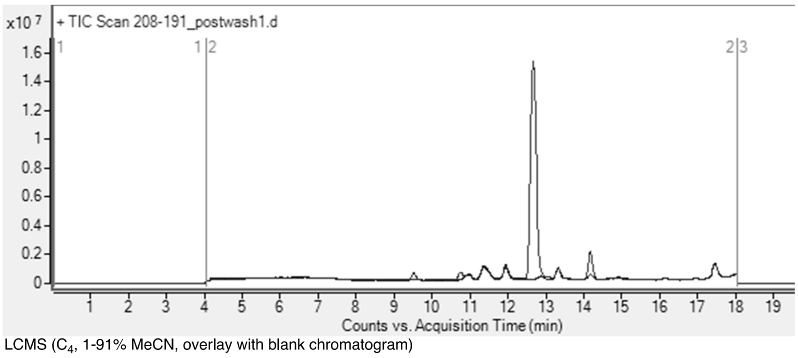

LCMS (C_4, 1-91% MeCN, overlay with blank chromatogram)

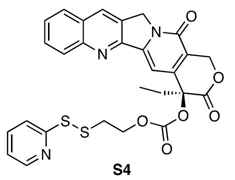

S4
$C_{28}H_{23}N_3O_6S_2$
M = 561.63 g/mol

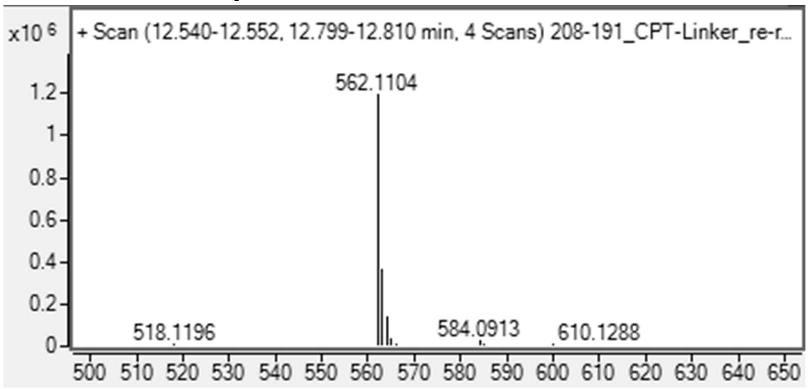

HRMS (ESI)

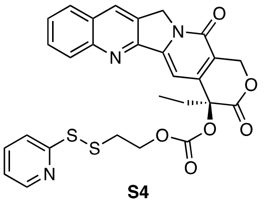

S4
$C_{28}H_{23}N_3O_6S_2$
M = 561.63 g/mol

Sequences and chromatograms. Amino acid sequences, as well as LCMS TICs or HPLC chromatograms and mass spectra of all proteins and peptides used in this study are depicted below.

BTP-7-CPT (S6)

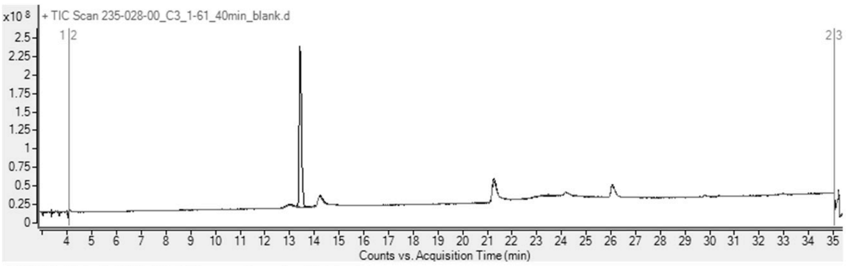

LCMS (C4, 1-61% MeCN, overlayed with blank)
BTP7-Campthotecin
S6
$C_{76}H_{103}N_{19}O_{18}S_2$
M = 1634.90 g/mol

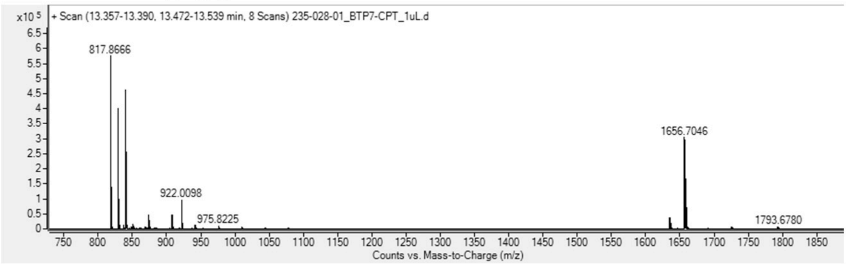

HRMS (ESI)
BTP7-Campthotecin
S6
$C_{76}H_{103}N_{19}O_{18}S_2$
M = 1634.90 g/mol

Scramble Scr-7-CPT (S8)

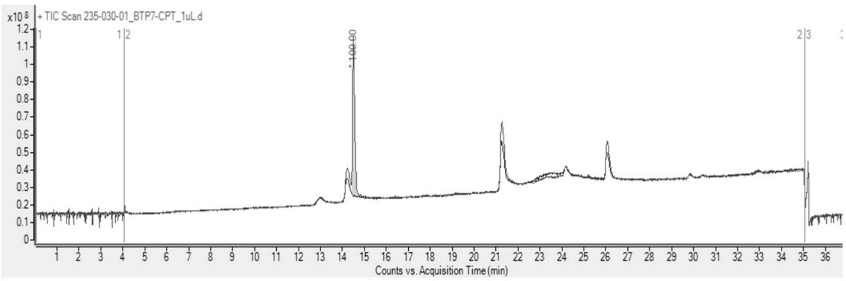

LCMS (C4, 1-61% MeCN, overlayed with blank)
Scrambled BTP7-Campthotecin
S8
$C_{76}H_{103}N_{19}O_{18}S_2$
M = 1634.90 g/mol

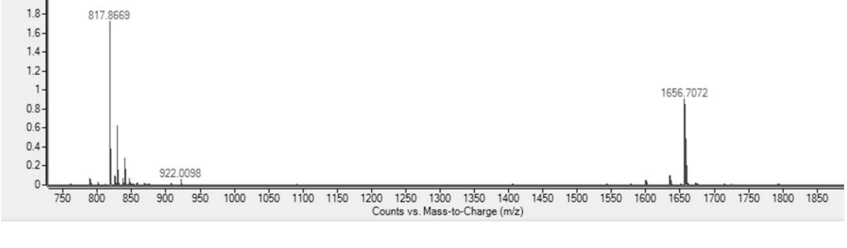

HRMS (ESI)
Scrambled BTP7-Campthotecin
S8
$C_{76}H_{103}N_{19}O_{18}S_2$
M = 1634.90 g/mol

Peptide: BTP-7-X (X = aminohexanoic acid)

Sequence: tkwGhvnk-X
Mass expected: 1080.6 Da
Mass observed: 1080.6 Da

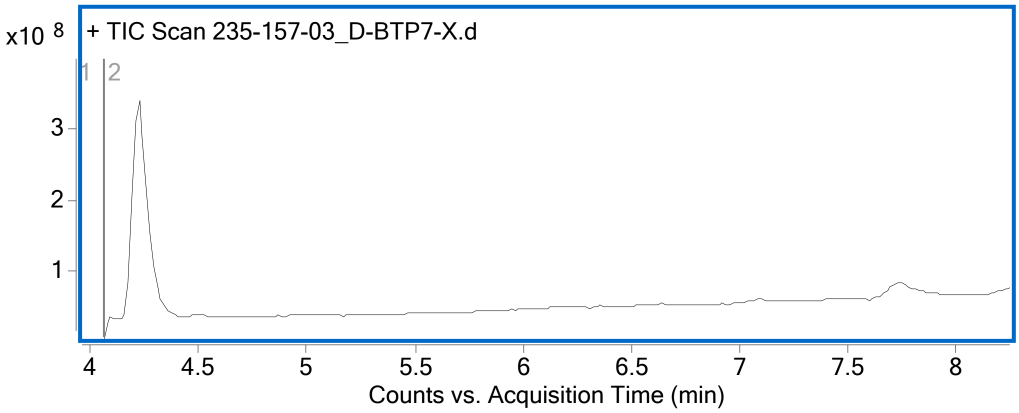

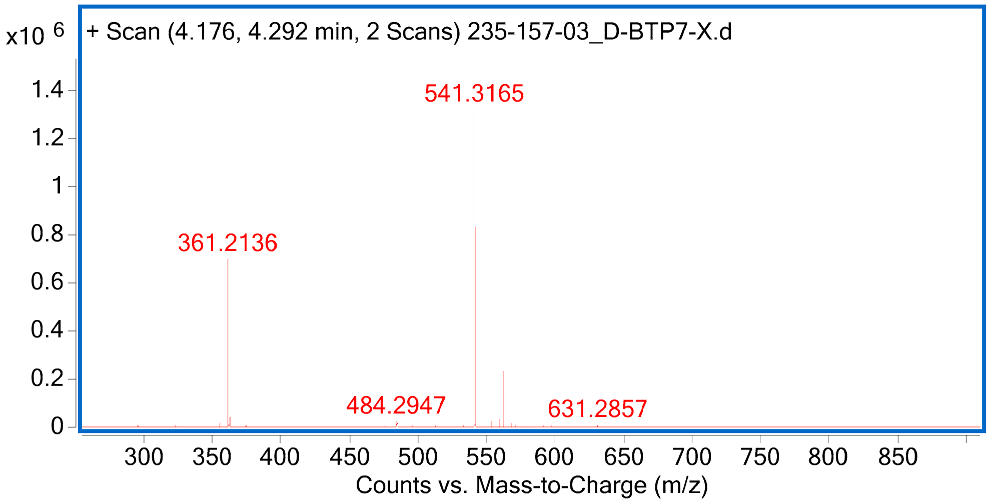

Peptide: BTP-7-X-C (X = aminohexanoic acid)

Sequence: tkwGhvnk-XC
Mass expected: 1183.6 Da
Mass observed: 1183.6 Da

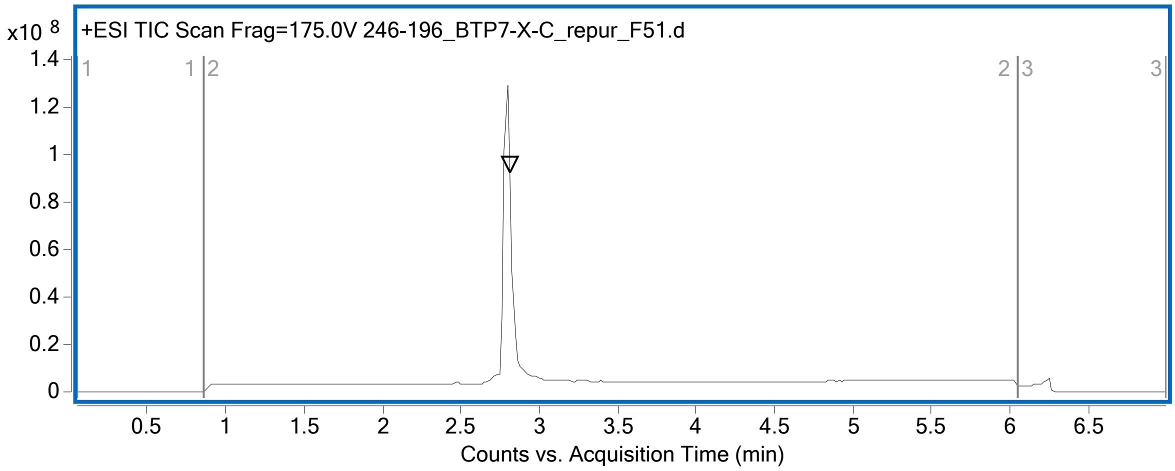

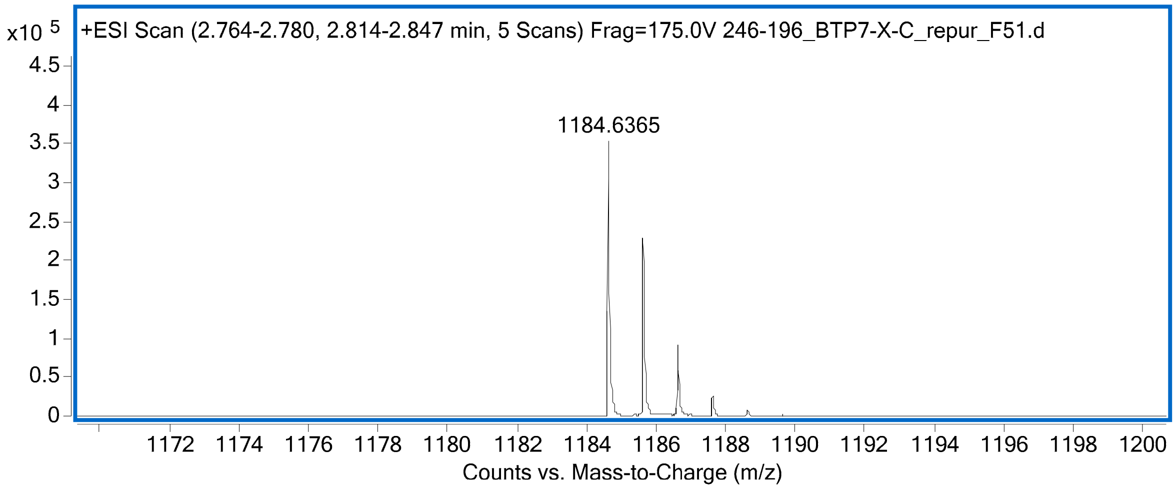

Supplementary Full Western Blots

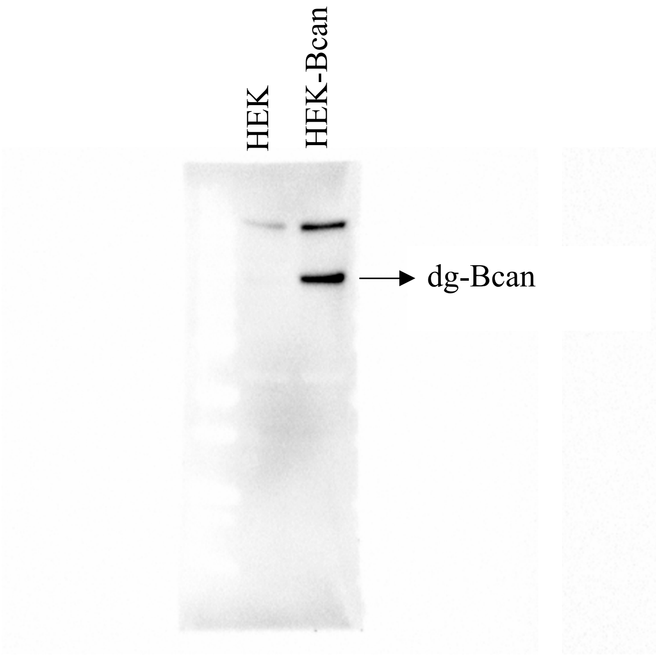

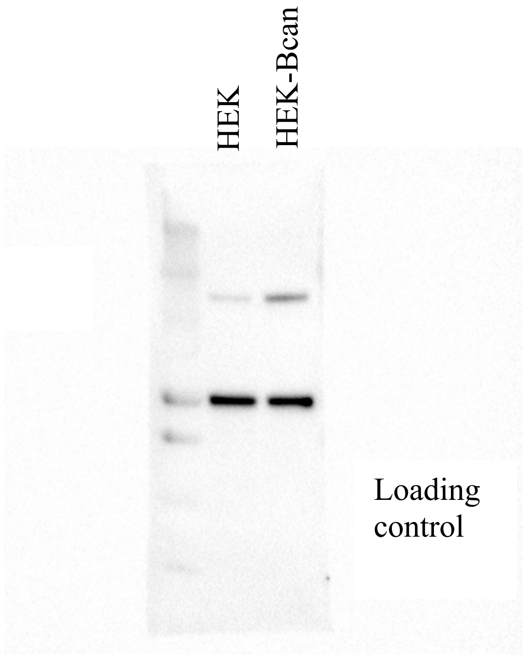

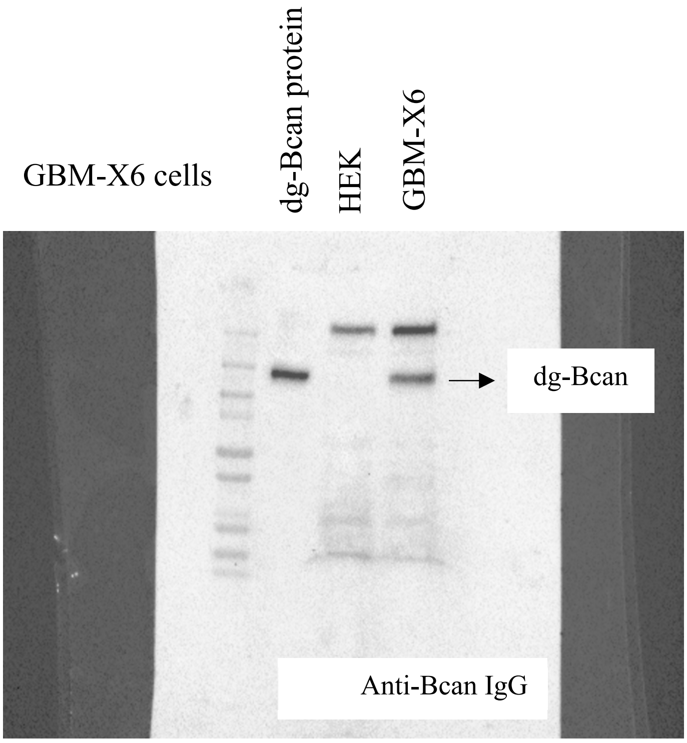

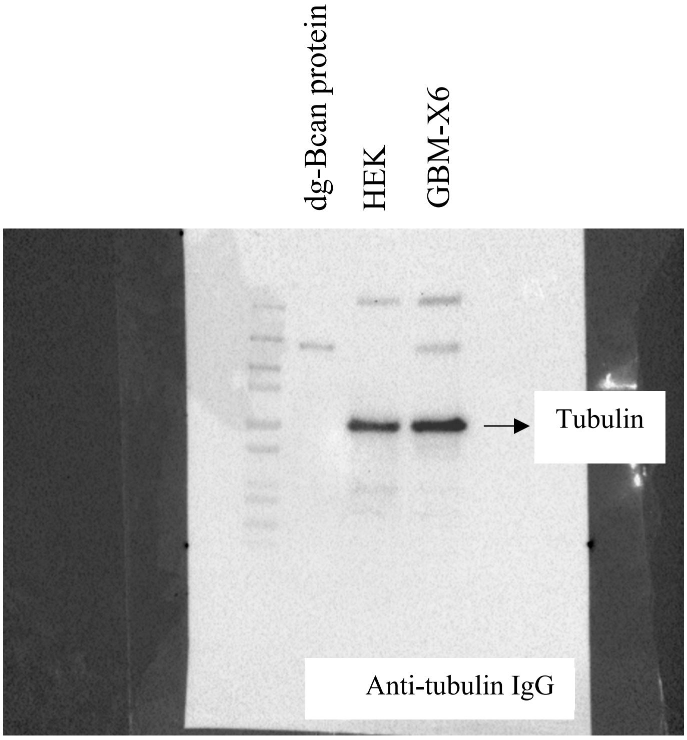

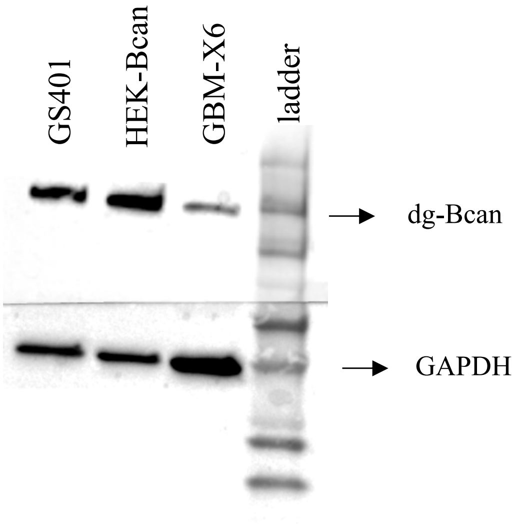
GS401
HEK-Bcan
GBM-X6
ladder
dg-Bcan
GAPDH
